# Embodying Control in Soft Multistable Robots from Morphofunctional Co‐design

**DOI:** 10.1002/advs.202503206

**Published:** 2025-07-30

**Authors:** Juan C. Osorio, Jhonatan S. Rincon, Harith Morgan, Andres F. Arrieta

**Affiliations:** ^1^ School of Mechanical Engineering Purdue University West Lafayette IN 47907 USA

**Keywords:** embodied control, inverse design, mechanical intelligence, modeling, multistability

## Abstract

Soft robots are distinguished by their flexibility and adaptability, allowing them to perform nearly impossible tasks for rigid robots. However, controlling their behavior is challenging due to their nonlinear material response and infinite degrees of freedom. A potential solution to these challenges is to discretize their infinite‐dimensional configuration space into a finite but sufficiently large number of functional modes with programmed dynamics. A strategy is presented for co‐designing the desired tasks and morphology of pneumatically actuated soft robots with multiple encoded stable states and dynamic responses. This approach introduces a general method to capture the soft robots' response using an energy‐based analytical model, the parameters of which are obtained using Recursive Feature Elimination. The resulting lumped‐parameter model enables the inverse co‐design of the robot's morphology and planned tasks by embodying specific dynamics upon actuation. This approach's ability to explore the configuration space is shown by co‐designing kinematics with optimized stiffnesses and time responses to obtain robots capable of classifying the size and weight of objects and displaying adaptable locomotion with minimal feedback control. This strategy offers a framework for simplifying the control of soft robots by exploiting the mechanics of multistable structures and embodying mechanical intelligence into soft material systems.

## Graphical abstract

A framework is introduced for co‐designing the morphology and planned tasks of soft robots by embodying specific reconfiguration modes and dynamics upon actuation, leveraging an efficient energy‐based analytical model and optimization. Desired kinematics are co‐designed with optimized stiffnesses and time responses to obtain robots capable of classifying the size and weight of objects and displaying adaptable locomotion with minimal feedback control.

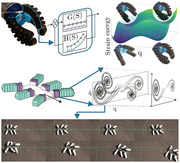

## 1 Introduction

Soft robots are characterized by their ability to interact with their environment, adapt to external stimuli, and protect against external disturbances.^[^ ^1^, ^2^, ^3^ ^]^ The inherent safety of soft robots stems from using low modulus of constituent materials, allowing them to perform tasks that are nearly impossible to their rigid counterparts ^[^ ^4^ ^]^. The interplay between soft mechanics and controls intrinsic to soft robotics gives rise to innovative solutions for executing tasks ranging from simple grasping to complex robot locomotion.^[^ ^5^, ^6^, ^7^, ^8^ ^]^ However, this interplay also poses challenges complicating their modeling and control. These challenges include their infinite dimensionality, material nonlinearity, and large deformations that most soft robots exhibit.^[^ ^9^ ^]^ As a result, sensory systems^[^ ^10^ ^]^ and complex predictive tools^[^ ^11^, ^12^ ^]^ are required to implement open‐loop control, leading to computationally demanding models to represent the robot's behavior. Embedding sensing and control in the robots' architecture^[^ ^13^ ^]^ offers a new strategy to address some of these challenges, whereby their intrinsically rich mechanical behavior is leveraged to reduce the modeling and control effort. For example, granular material‐based universal grippers^[^ ^14^ ^]^ can adapt to and grasp different object shapes without needing closed‐loop control based on their mechanical response. Zoe et al.^[^ ^15^ ^]^ developed a methodology to use various soft actuators as pneumatic sensors, enabling distance and shape sensing, profile scanning, and stiffness determination. Other approaches focused on using mechanical instabilities for sequential programming and fast actuation. Yang et al.^[^ ^16^ ^]^ explore the sequential programming of a soft gripper to perform grasping and twisting motions driven solely by mechanical instabilities in the gripper's body. Another relevant example by Lue Y. et al.^[^ ^17^ ^]^ demonstrates the use of snap‐through instabilities for fast actuation and reconfiguration. These examples illustrate how the intelligent co‐design of the robot's body morphology and functional behavior enables an approach to close the control loop for realizing desired tasks.

Multistable structures offer an alternative path to achieve control of soft robotics with feedback via the programming of input‐specific stable and defined shapes.^[^ ^18^, ^19^, ^20^ ^]^ These structures display multiple energetically favorable configuration, enabling a soft system to reach different final shapes from pre‐defined actuation inputs. Multistable structures are often the result of assembling multiple classical bistable sub‐structures, including constrained beams/trusses,^[^ ^21^, ^22^ ^]^ constrained dielectric elastomers,^[^ ^23^ ^]^ shells,^[^ ^18^, ^24^, ^25^, ^26^ ^]^ compliant mechanisms,^[^ ^27^ ^]^ or inflatable structures.^[^ ^28^ ^]^ Presently, a wide variety of soft machines leverage mechanical instabilities to improve their design and performance^[^ ^29^, ^30^, ^31^, ^32^, ^33^, ^34^, ^35^ ^]^ by programming different stable configurations and utilizing the rapid energy release during a snap‐through instability.^[^ ^17^, ^36^, ^37^, ^38^, ^39^ ^]^ Conrad et al.^[^ ^40^ ^]^ show an electronics‐free controller for a complex pneumatic system based on soft logic gates that can be integrated into different soft machines. Similarly, Peretz et al.^[^ ^41^ ^]^ utilized mechanical instabilities to create fluidic logic and reproduce valving functionality using entirely soft elements. Melancon et al.^[^ ^42^ ^]^ used a bistable origami pattern to program different target points to its actuator, achieved by a single pressure source and predefined pressure path. Van Raemdonck et al.^[^ ^43^ ^]^ geometrically tuned an actuator to generate complex actuation sequences from a single input by delaying the unit's snap‐through. These examples show the versatility of multistable structures in soft robotic systems, as the robot's configuration in response to actuation inputs can be predicted based solely on its structural response. Consequently, by transforming the infinite‐dimensional deformation space into a finite number of stable states, the complexity of predicting the soft robot behavior is reduced, and its feedback can be expressed as a set of available stable configurations. This approach can simplify the complexity of predictive or data‐driven algorithms currently needed to design and control soft robots, ultimately making soft robots more accessible and practical. However, the full potential of using multistability and programmed mechanical responses for soft robotics can only be realized by establishing computationally efficient methodologies for their morphological and functional co‐design that circumvent the long runtime roadblocks imposed by the predominant use of conventional Finite Element (FE) packages to predict the geometry and response characteristics associated with the desired stable states.

**Figure 1:**
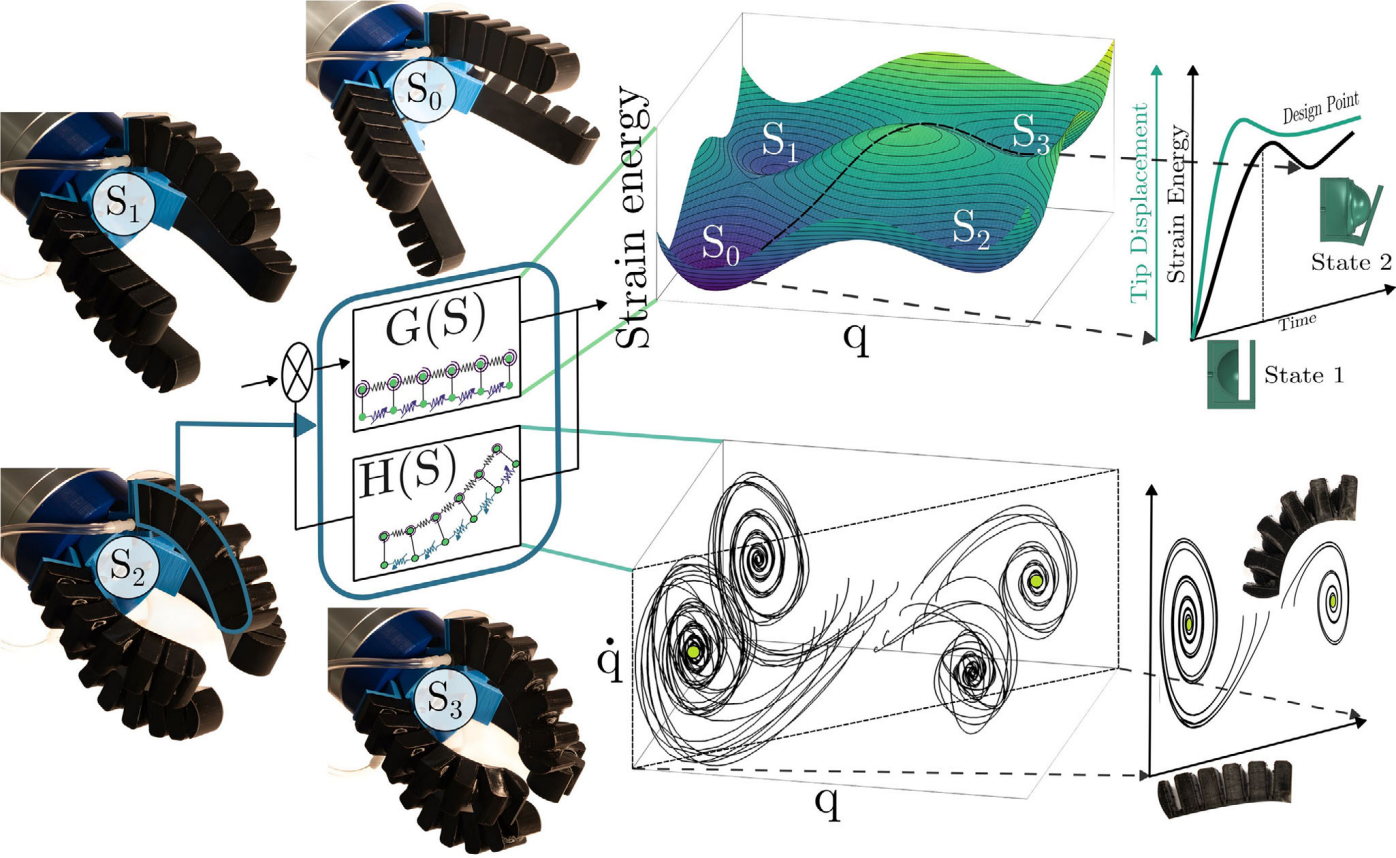
Multistable soft robot with pre‐programmed dynamics for different stable positions and stiffnesses. Our robot displays four grasping set points (*s*_0_, *s*_1_, *s*_2_, *s*_3_) and embodied/morphological control. The robot's morphology and mechanical behavior stems from the resulting energy landscape, which encodes desired input and output dynamics (G(s)). Feedback H(s) is derived from the dynamic response of the robot, whereby designed attractors for each stable set point are encoded in the robot geometry. The system's output shows four set points as static minima in the energy landscape and attractors in the phase portrait, where *q* and $\dot{q}$ are a schematic representation of the robot's generalized coordinates. Set points (energy minima) are accessed by activating/inverting the dome units: State 1 → Stress‐free state and State 2 → Inverted state.

We introduce a framework for the modeling and inverse design of soft robotics with encoded mechanical behaviors by mapping into a discretized space the desired kinematic configurations leveraging mechanical instabilities (see **Figure** **1**). We achieve this by integrating different dome‐shaped, programmable units addressable via pneumatic actuation featuring bistable, pseudo‐bistable (metastable), and monostable^[^ ^24^, ^44^ ^]^ mechanical behaviors (see Figure S2, Supporting Information). As a result, different set points in the form of specific kinematical shapes and time responses are embodied into the system's morphology (Figure 1), giving our soft robots the inherent capability of reaching different pre‐programmed positions and performing specific dynamic behaviors (i.e., grasping timing and release, and locomotion) under open‐loop inputs with almost zero error. This characteristic allows for robust task design of our soft robots, as the bistable units provide intrinsic kinematic control dictated by the embodied mechanical response. The position control inherent to the designed multistable morphology can serve as set points for triggering different control actions, decoupling the actuation from the robot's final configuration (i.e., shape). At the same time, specific dynamic responses can be programmed along each set point to trigger desired behaviors. The discretized response afforded by the proposed topology enables the introduction of an energy‐based analytical modeling framework, allowing the efficient shape and structural property prediction of different multistable soft robots that utilize the dome shape unit topology as a building block (in our case, the Dome Phalanx Finger ‐ DPF) that ultimately enables the inverse co‐design of the robot's functionality and control via the morphology. Our modeling approach is based on lumped parameter elements that can capture different mechanical behaviors of the dome unit via mechanics‐informed identification, mapping the geometry of our robot to its mechanical response. The collective interaction of these elastic elements dictates the soft robot's configuration, stiffness, and dynamic response. We demonstrate our approach's capability for inverse co‐design of DPF‐based robots by programming targeted static shapes with maximal stiffnesses and desired dynamic behaviors leveraging viscoelastic effects. Ultimately, the static and dynamic response co‐design allowed us to embody functional behaviors, including object classification, pre‐programmed pick‐and‐place tasks, and the controlled locomotion of a six‐legged soft walker. The presented modeling and inverse co‐design strategy enables a path for encoding an electronics‐free form of mechanical intelligence that simplifies the actuation and control of soft robots with implications on their accessibility, recyclability, security, and cost.

## 2 Results

### 2.1 Embodied Position and Stiffness Control

**Figure 2:**
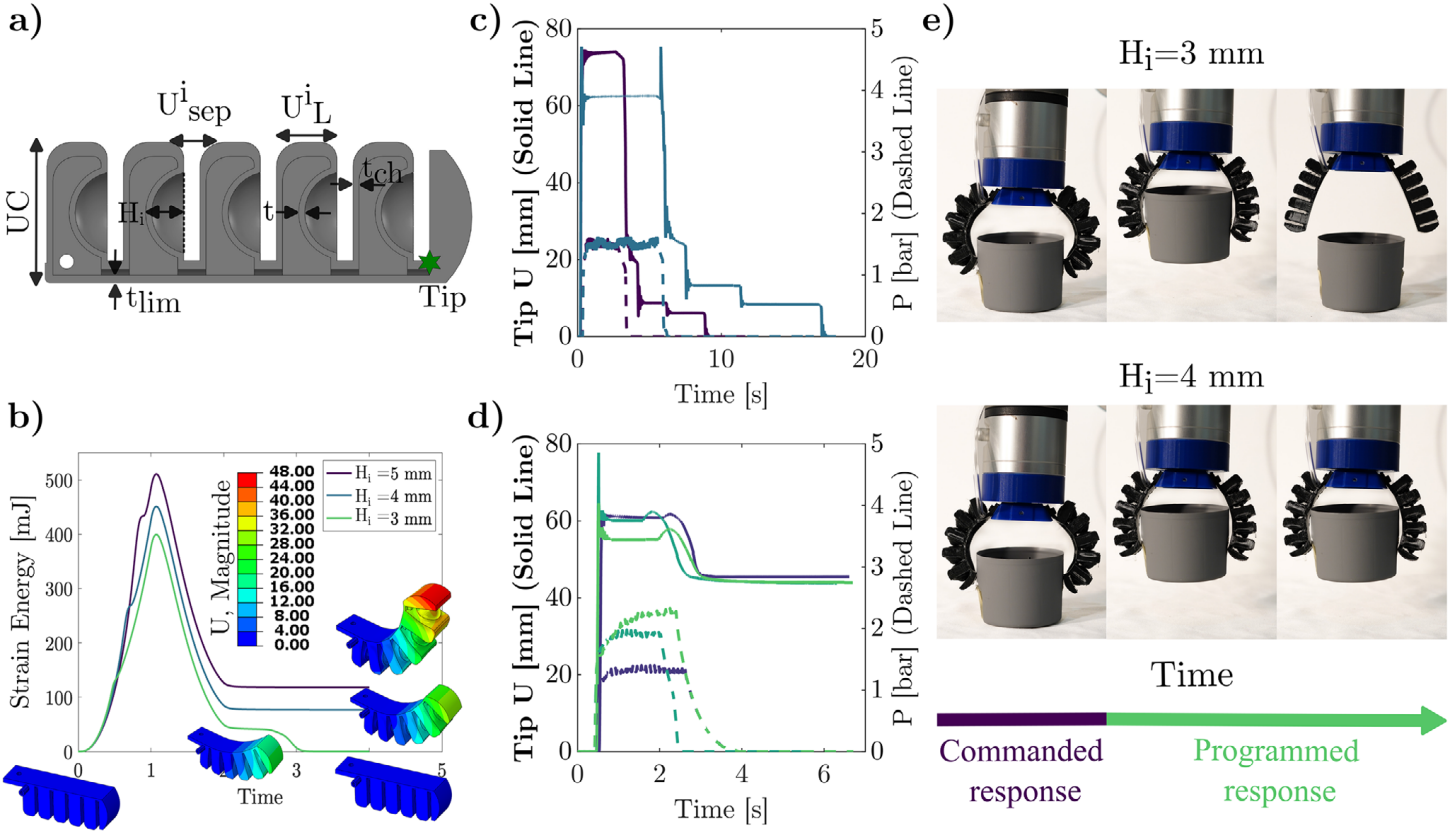
Dome Phalanx Finger (DPF) with different encoded mechanical responses and behaviors. a) 5‐segment DPF geometry parameters (*i* = 5). b) Effect of dome height on finger response (Every segment with the same height). *H*_*i*_ = 5 mm and *H*_*i*_ = 4 mm show a bistable behavior, and *H*_*i*_ = 3 mm shows a pseudo‐bistable (metastable) response (self‐resetting system). c) Metastable finger (*H*_*i*_ = 3 mm) Tip displacement and pressure over time. d) Bistable finger (*H*_*i*_ = 5 mm) tip displacement and pressure over time. The same final position is achieved despite different actuation magnitudes (Input pressure). e) Time lapse of two gripper architectures showing both bistable and pseudo‐bistable responses.

We begin by modifying the common PneuNet bending actuator topology^[^ ^45^ ^]^ via the inclusion of dome‐shaped units to create our DPF topology (see **Figure** **2**a). The finger derives its behavior from the mechanical response of the dome‐shaped shell elements and the interaction between each segment. As the units invert, the top section of the finger expands, while the limiting layer (see Figure S1d, Supporting Information) retains its original length. This results in global curvature and different final stable shapes, depending on the number of inverted dome units (see Figure S2b, Supporting Information). These dome‐shaped units program different mechanical responses and stable shapes into the system, effectively discretizing the infinite‐dimensional deformation space into a manageable number of kinematic configurations dependent on the dome unit geometry, each attainable via well‐defined, open‐loop inputs. The mechanical response and stable configurations of the DPF can be tuned by adjusting the dome height per unit (*H*_*i*_), dome thickness (*t*), number of segments, dome unit size (UC), pneumatic chamber thickness (*t*_ch_), limiting layer thickness (*t*_lim_), dome unit length ($U_{L}^{i}$), air chamber dimensions (*W*_ch_ and *t*_mid_), and the spacing between adjacent cells per unit ($U_{\text{sep}}^{i}$) (see Figure 2a; Figure S1a, Supporting Information, for reference). The geometric parameters of the dome units *H* and *t* dictate the mechanical response, determining whether it is monostable (see Figure S1c(i)), metastable (see Figure S1c(ii) and Movie S1, Supporting Information), or bistable (see Figure S1c(iii), Supporting Information). Furthermore, the transient response of the DPF can be controlled by the material properties and additional geometric parameters (*t*_lim_, *t*_ch_, *t*_mid_), which significantly influence global curvature, system overshoot, and steady‐state error (see Figure S3, Supporting Information).

Given the broad range of geometrical configurations, each finger can be geometrically tuned to exhibit different mechanical responses: a bistable response, where the final shape is retained after dome inversion^[^ ^18^, ^24^, ^46^ ^]^ (Figure 2b *H*_*i*_ = 4 mm and *H*_*i*_ = 5 mm). A metastable response, where each unit undergoes snap‐through adopting a shape that is retained for a time *t* = τ after the load is removed, before returning to its stress‐free state as a consequence of the combined effects of the unit's viscoelastic material behavior and dome geometry^[^ ^36^, ^47^, ^48^ ^]^ (Figure 2b, *H*_*i*_ = 3 mm; Movie 1, Supporting Information). Lastly, a monostable response, where the material behavior dominates over the geometric response and the finger responds similarly to a conventional PneuNet bending actuator. As a result of the encoded mechanical responses, the actuation pressure required to invert the dome units is decoupled from the final shape, which is entirely determined by geometric parameters (see Figure 2d). Moreover, we leverage the material's viscous response and structural properties to control the reset dynamics of the DPF (see Figure 2c), by controlling the unit metastable behavior (dependent on dome height, material properties) and the duration of the applied load. The resulting freedom to design the DPF's dynamics and set points allows for pre‐programming a diverse set of tasks, sequences, and positions.

To showcase these unique capabilities, we developed a two DPF gripper architecture. By changing the dome height, we can program a gripper composed of two fingers to pick and hold an object after actuation (Figure 2e, *H*_*i*_ = 4 mm) or pick an object and release it as the dome units reset (Figure 2e, *H*_*i*_ = 3 mm), the latter of which is ideal for embodying different dynamic response dependent on the robot morphology. The number of stable states that can be encoded using the DPF morphology equals the number of constitutive units where each unit is tunable independently. Consequently, different unit geometries can be combined to achieve multiple stable states, enabling a diversity of behaviors with the same overall finger topology (Bistable + Metastable DPF). This allows for different energy minima and time responses to be embodied within the same finger, which can be accessed by a single pressure input and serve as discrete and functional robot configurations.

### 2.2 Model Derivation from Mechanics‐Informed Parameter Determination

**Figure 3:**
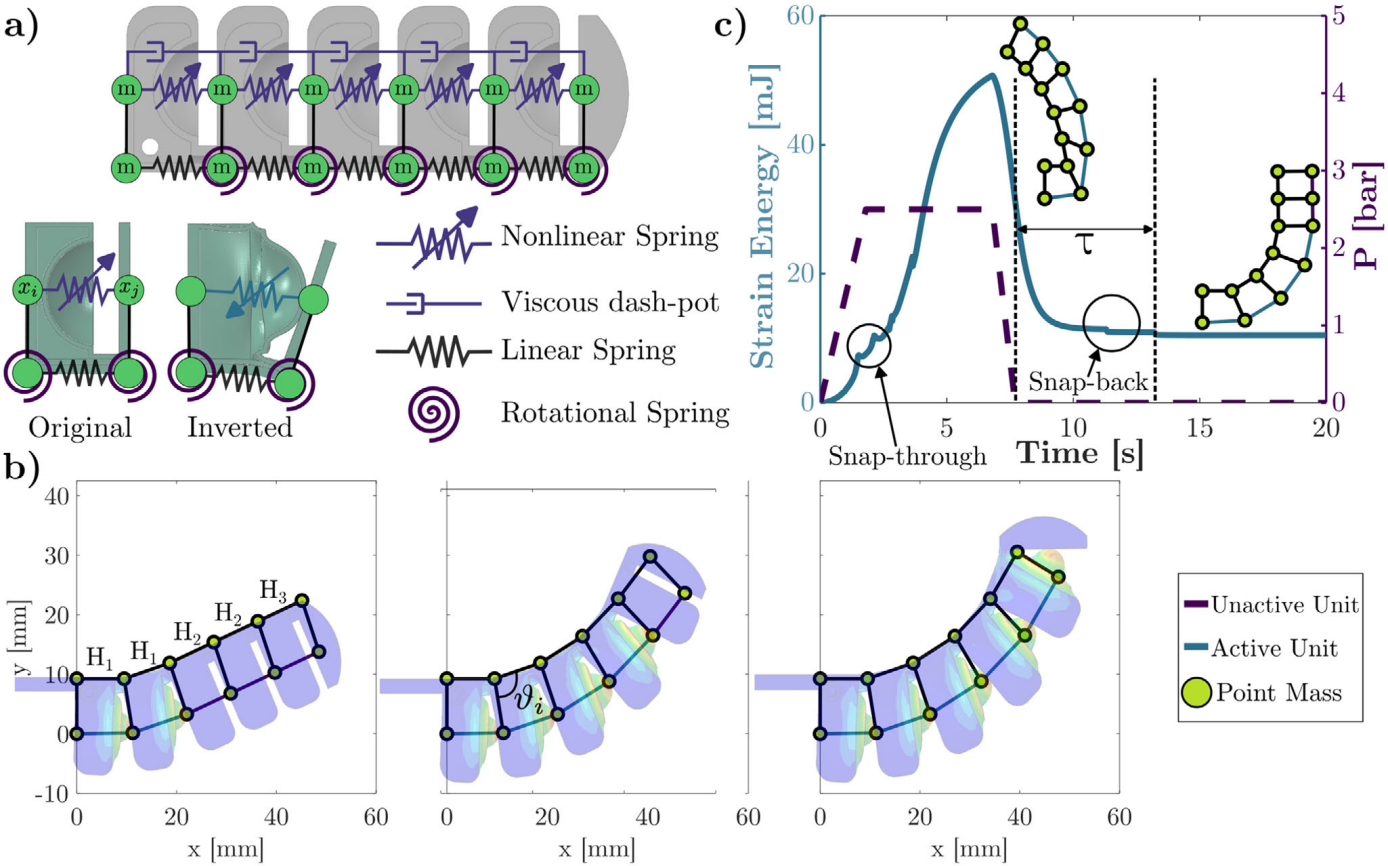
An energy‐based model for static and dynamic analysis of multistable soft robots based on the DPF topology. a) Spring lattice model with linear, rotational, and nonlinear springs. b) Static stable states predicted by the lattice model for *H*_1_ = *H*_2_ = 4 mm, *H*_3_ = *H*_4_ = 4.5 mm, and *H*_5_ = 5 mm. c) Dynamic response for the soft robot with four bistable units and two metastable units (*H*_1_ = *H*_2_ = *H*_3_ = *H*_4_ = 5 mm, and *H*_5_ = *H*_6_ = 3 mm). The model captures each bistable unit's snap‐through and metastable units' snap‐back (See Movie S2, Supporting Information).

A simple yet reliable modeling framework is necessary to design, combine, and predict the finger's behavior based on its geometry. Given the broad design space and the computational cost of using FE models for our finger topology, we propose an energy‐based spring model that captures the complex mechanical behaviors needed to design multistable soft robots with desired functional characteristics. Establishing an analytical model to capture the global behavior of the DPF requires consideration of both the unit cells' and the global geometric parameters. By capturing the contribution of each constitutive unit of the DPF and their interactions, we can construct an energy landscape with a simplified model, where each of the minima corresponds to a stable state and a final position of the system. In this way, the system's state (*x*_*i*_) can be extracted from the resulting strain energy potential via a minimization process, whereby the DPF's geometrical and stiffness characteristics dictate the programmed stable shapes. The DPF geometry is represented by a lattice array comprising nonlinear, linear, and torsional springs for the static response and point masses and dash‐pots for the dynamic response (**Figure** **3**a). The energy for the springs used in the lattice can be written as follows:^[^ ^49^ ^]^

(1) $$\begin{array}{cc} & \text{Nonlinear:}\;E_{NL}\left(x_{ij}\right)=\frac{1}{2}k_{b}{\left(\left|\right|x_{ij}\left|\right|-s_{ij}\right)}^{2}\\ & \;\times \left(1+\left(1-\alpha \right)\left({\left(\frac{\left(\left|\right|x_{ij}\left|\right|-s_{ij}\right)}{d}\right)}^{2}-2\frac{\left(\left|\right|x_{ij}\left|\right|-s_{ij}\right)}{d}\right)\right) \end{array}$$

(2) $$\begin{array}{cc} & \text{Linear:}\;E_{L}\left(x_{ij}\right)=\frac{1}{2}k_{l}{\left(\left|\right|x_{ij}\left|\right|-s_{ij}\right)}^{2} \end{array}$$

(3) $$\begin{array}{cc} & \text{Torsional:}\;E_{T}\left(\vartheta \right)=\frac{1}{2}k_{\vartheta }{\left(\vartheta -\vartheta_{0}\right)}^{2} \end{array}$$

(4) $$\begin{array}{cc} & E\left(x_{ij}\right)=\sum_{i=1}^{n}E_{L}\left(x_{ij}\right)+E_{NL}\left(x_{ij}\right)+E_{T}\left(\vartheta \right). \end{array}$$

where *x*_*ij*_ = *x*_*i*_ − *x*_*j*_, ϑ is the angle between the rigid link and the limiting layer (see Figures S5a and S7c, Supporting Information), ϑ_0_ is the initial angle between the rigid link and the limiting layer, and *k*_*b*_, *k*_*l*_, *k*_ϑ_ are stiffness parameters that depend on the DPF geometry. The individual stiffness and spring connectivity allow us to capture the limiting layer and dome inversion effects. We use a nonlinear spring to represent different dome mechanics (Figure S1c, Supporting Information), which are described by the parameters *k*_*b*_, α, and *d*. Linear and rotational springs are utilized to capture the limiting layer of the finger by combining stretching and bending energies (see Figure 3a). The connection between the limiting layer and the nonlinear springs is modeled as an infinitely rigid connection, given that the strain is negligible compared to the rest of the lattice elements. By minimizing the sum of all contributions to the system (Equation 4), the static problem is solved, and all possible finger stable configurations are predicted (see Figure 3b).

A primary challenge of spring‐lattice models is to accurately map the geometric parameters of the system to its stiffness constants. To address this challenge, we integrate FE simulations (see Section A.2, Supporting Information) with Recursive Feature Elimination (RFE; see Section A.3, Supporting Information)^[^ ^50^ ^]^ and lasso regression to derive expressions for *k*_*b*_, α, and *d*–the nonlinear spring parameters in Equation 1–as functions of dome height (*H*), dome thickness (*t*), dome curvature (*R*) and Young Modulus (*E*) of a generic constitutive unit (see Figure S1, Supporting Information). We considered different dimensionless relations (π_*i*_) based on the most relevant geometric interactions affecting the dome's energy.^[^ ^24^ ^]^ These mechanics‐informed interactions enhance the generality of the analysis by linking the mechanics to the unit's geometry. For our model, we assume all spring constants can be expressed as functions of the shell's load‐carrying capacity ($\pi_{1}=\frac{t}{H}$), curvature‐to‐thickness ratio ($\pi_{2}=\frac{t}{R}$), and dome shallowness ($\pi_{3}=\frac{H}{R}$). Consequently, each parameter can be formulated as:

(5) $$k_{b}=C\left(\pi_{1},\pi_{2},\pi_{3}\right)\xi_{k}\;\alpha =C\left(\pi_{1},\pi_{2},\pi_{3}\right)\xi_{\alpha }\;d=C\left(\pi_{1},\pi_{2},\pi_{3}\right)\xi_{d}$$

where **C**(π_1_, π_2_, π_3_) is a matrix containing all possible candidates and interactions of the non‐dimensional relations, as shown in Equation S.13 (Supporting Information). Using RFE, the relevance of each feature can be automatically determined by iteratively removing one feature at a time and observing the model's coefficient of determination (*r* ^2^). This process yields the weights ξ_α_, ξ_*k*_, and ξ_*d*_ with fewer features while maintaining high accuracy. Notably, only the unit cell is used to determine the parameter features; consequently, the DPF's mechanical behavior is derived. A wide range of Young's modulus (*E*), dome height‐to‐dome base ratio $\left(\frac{H}{r_{b}}\right)$, dome base radius (*r*_*b*_), and thickness (*t*) are simulated to capture the entire design space of the DPF, including various soft materials (5MPa ⩽ *E* ⩽ 40 MPa). As a result, we derive a general equation in terms of the three non‐dimensional parameters (Equation S.15, Equation S.16, and Equation S.17, Supporting Information; see Section A.3.3, Supporting Information, for details). Using these derived expressions and a gradient‐based optimization algorithm (see Section A.4, Supporting Information, for details), the final shape of the finger can be predicted by minimizing the system's energy, which is now expressed as a function of the geometric parameters of the structure.

A comparison between FE simulations and the proposed model for different stable states of the finger (see Figure 3b) and different design cases (see Figure S10, Supporting Information) shows good agreement with the energy‐based lattice model while also reducing computational time by more than three orders of magnitude (see Table S2, Supporting Information).

The model is further extended to capture the structure's dynamic response, allowing us to observe the material's viscoelastic behavior and analyze metastable cases that energy minimization alone cannot predict. The dynamic response for different unit cell geometries is shown in Figure S7b (Supporting Information), and for different five‐segmented fingers in Figure S7d (Supporting Information), where the dynamic model predicts the same final state of the system after reaching a steady state. To explore the combined behavior of different dome geometries, we modeled a five‐segment DPF topology with bistable and metastable units (see Figure 3c). We stacked four bistable units (*H*_1_ = *H*_2_ = *H*_3_ = *H*_4_ = 5 mm) and two metastable units with the same dome height (*H*_5_ = *H*_6_ = 3 mm). This configuration allows us to achieve one unique set point: a stable state and a time‐dependent state that holds its shape for a given predictable time τ (see Figure 3c). The resulting dynamic response features five different snap‐through events and two snap‐backs (dome‐unit resets), adequately representing the complex dynamics displayed by the DPF (Figure 3c).

### 2.3 Inverse Co‐Design

Based on the model's accuracy and agreement with FE simulations, we formulate an inverse problem to co‐design diverse DPF topologies capable of reaching a desired pre‐programmed set point and dynamic behavior. Since the system's stability is governed by its stable states, the optimization algorithm searches only within feasible stable configurations (i.e., the number of active units) and their neighborhoods, resulting in high computational efficiency. To this end, we employ Bayesian optimization^[^ ^51^ ^]^ to explore various design parameters, including the number of segments, dome height for each segment (*H*_*i*_), dome thickness (*t*), unit separation for each segment ($U_{\text{sep}}^{i}$), and unit length for each segment ($U_{L}^{i}$) (See Figure 2a for parameter definitions and Figure S11, Supporting Information, for the design space). We consider different objective functions to demonstrate the versatility of the DPF topology in encoding distinct final positions and dynamic responses with minimal actuation. First, we target a specific tip position while simultaneously maximizing stiffness (Position + Stiffness Design; see Section A.6, Supporting Information), thereby enabling embodied control with maximum stiffness. Second, we exploit the dynamic response of the structure to encode task‐specific behaviors directly into the DPF topology, enabling task planning capabilities.

**Figure 4:**
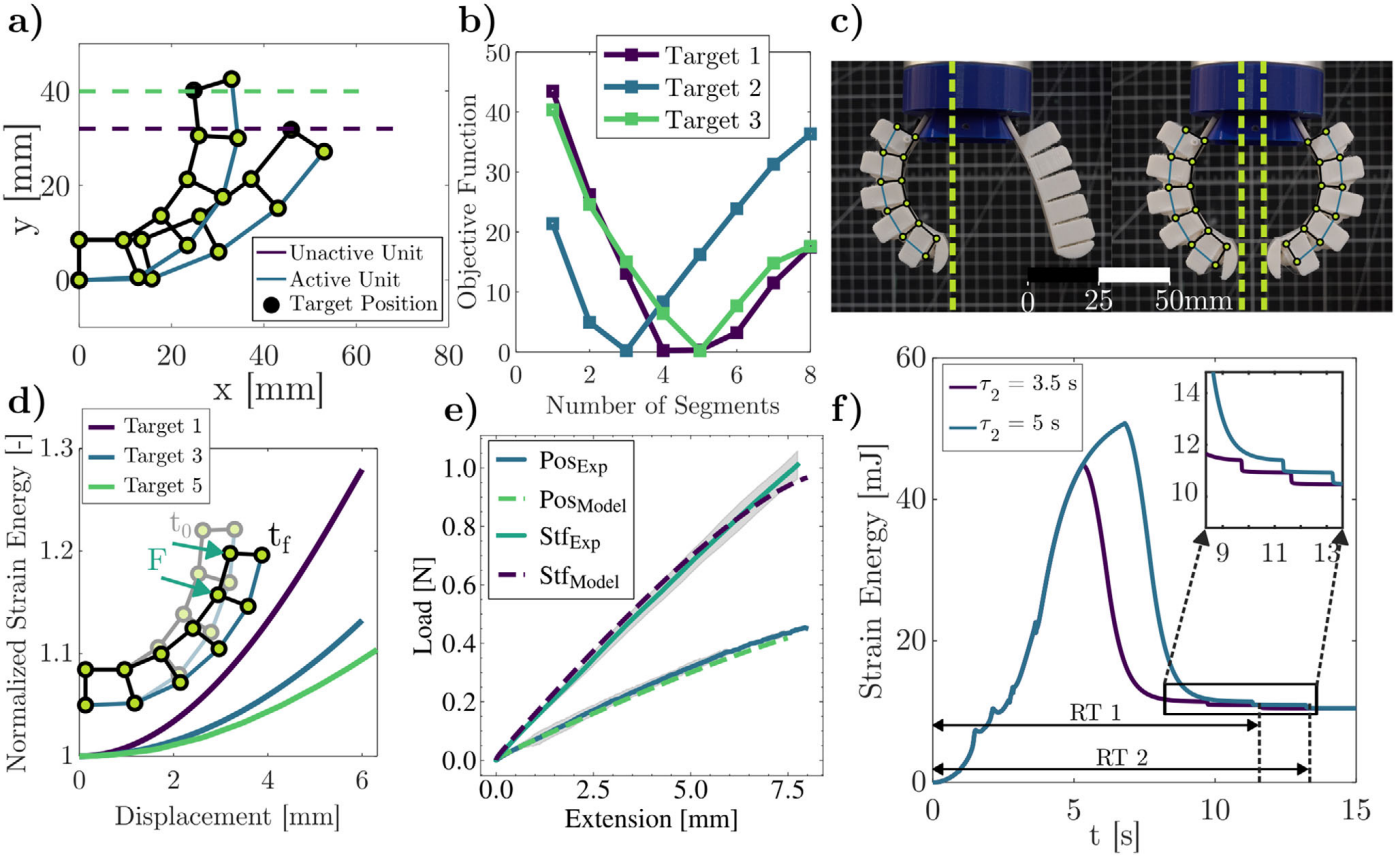
DPF inverse design and experimental validation. a) Predicted results for Target 1 and 3 from the optimization model (Position Design). b) Objective function values as a function of the number of segments, with the minimum value identified for each target position. c) The gripper achieves multiple stable kinematical states predicted by the energy‐based model, which are successfully transferred to 3D‐printed prototypes. d) Stiffness determination of the DPF using a follower force approach, with normalized strain energy vs. displacement curves, is shown for Objectives 1, 3, and 5. e) Comparison of the proposed model and experimental results for position‐only (Pos) optimization and combined position + stiffness optimization (Stf). f) Dynamic response for a six‐section DPF under two different loading profiles.

**Position + stiffness design**:

Given a target tip coordinate [x,y] (Target_*xy*_), the inverse problem objective function can be written as:

(6) $$\begin{array}{cc} & \min_{H_{i},t,{\text{Unit}}_{L}^{i},{\text{Unit}}_{s}^{i}}w_{1}{\left({\text{Target}}_{\mathrm{xy}}-{\text{Tip}}_{\;\text{dis}}\right)}^{2}+w_{2}{\left(\frac{1}{dF_{\text{in}}/\mathrm{dx}}\right)}^{2}\\ & \text{s.t.}\;H_{i+1}\leq H_{i},\;i=1,\text{…},N-1\\ & \;\;U_{\text{sep}}^{i+1}=U_{\text{sep}}^{i},\;i=1,\text{…},N\\ & \;\;U_{L}^{i+1}=U_{L}^{i},\;i=1,\text{…},N \end{array}$$

where Tip_dis_ represents the tip point (see Tip in Figure 2a), which coincides with the top node of the finger's last unit. *w*_1_ and *w*_2_ are the weights assigned to each objective, and *dF*_in_/*dx* is the stiffness obtained from the model (see details in section A.6.3, Supporting Information). A domain constraint is imposed to guarantee that every dome unit is higher than the one in front (*H*_*i* + 1_ ⩽ *H*_*i*_), which in some instances can lead to up to N possible stable states (N being the number of segments). Moreover, we impose constraints for all unit separations and unit lengths to be equal for simplicity and design domain to guarantee bistability on all units, based on the phase diagram shown in Figure S1b. However, different restrictions can be imposed depending on the purpose of the DPF design, and parameters can be individually tuned to achieve a large variety of positions and stable states (see Movie S3, Supporting Information). Our algorithm was tested by defining five different tip coordinate targets using two materials with distinct properties (NinjaFlex 85A and Cheetah 95A) optimized to achieve the desired positions. For each case, the target coordinates in *x* and *y* were provided to the algorithm (Target Position in **Figure** **4**a), and the optimization was performed by incrementally adding one unit per run and calculating the objective function. To reduce the computational cost, a maximum of 8 units was specified (Figure 4b), although additional units can be included if the objective requires a larger finger geometry. The geometry with the minimum objective function value (Obj in Tables S4 and S5, Supporting Information) was selected and plotted to verify whether the desired position was achieved (see Figure 4a). Geometric parameters, coordinate targets, and objective function values are reported in Tables S4 and S5 (Supporting Information). The final stable state and its comparison with each target can be observed in Figure S12 (Supporting Information). To validate our optimization algorithm, we 3D printed the five different geometries shown in Table S9 (Supporting Information) using both tested materials (NinjaFlex 85A and Cheetah 95A) and measured the final tip position after commanded dome inversion. An average error of 7.3% was measured across all samples, showing good agreement with the optimization algorithm and the lattice model. To evaluate the impact of including stiffness in the objective function, we experimentally tested (see Figure S15, Supporting Information) the five topologies generated via position‐only optimization (Equation S.30, Supporting Information) and position + stiffness optimization (Equation 6). Using this co‐design strategy, the DPF stiffness has increased on average 1.94 times for all the test cases, achieving maximum stiffnesses of 0.55 N/mm, 0.3 N/mm, and 0.15 N/mm for cases 2, 4, and 5, respectively (see Figure 4e for case 5), which consequently can enhances the achievable grasping force.

The topologies generated by this methodology exhibit deterministic behavior despite using polymeric materials, meaning that kinematic states are consistently attained as long as the gripper's geometric parameters remain unchanged (see Figure 4c; Movie S4, Supporting Information). Specifically, we cycle each gripper topology between the initial and fully activated states, verifying the gripper's ability to achieve the same aperture and positioning across multiple cycles (Tests 1–3 in Figure 4c). Furthermore, we assessed material and time degradation effects by performing 300 cycles over three weeks (see Figure S16, Supporting Information), which demonstrates that even after numerous activations and an extended period, the gripper's performance remains within 5% of the initial design behavior. This characteristic enables robust robot design, as the bistable elements provide intrinsic kinematic control dictated by the gripper's mechanical response. It is worth mentioning, that the pressure for activating the units is not controlled using sensors and closed‐loop control, illustrating the embodied self‐regulation from the designed multistability. Consequently, the position control inherent to the designed multistable structure serves as control set points that can be embodied in the robot's morphology and achieved with a single, open‐loop controlled actuator.

**Dynamic response design**: The unit cell morphology and co‐design approach also allow us to encode desired dynamics responses in our soft robots. To this end, we utilized the model described in the previous section and added a term to account for the resetting time. Using the dynamic model (see Section A.4, Supporting Information), the metastable units' resetting time is calculated, and the final position of the finger is determined. Given this, specific set points and dynamic behavior in the vicinity of that stable point can be designed. For this particular case, we selected a six‐segment DPF with fixed dome heights for the first four units, and we utilized our optimization approach to maximize the resetting time under two different configurations. It is worth mentioning that the rest of the geometric parameters of the DPF (*U*_*sep*_, *U*_*L*_, *t*_*lim*_) are kept constant during the optimization process, as their effect on the resetting time is negligible. At the same time, the optimization algorithm is utilized to determine the dome heights of the last two units and the remaining geometric parameters (*t*). Given this, the optimization problem can be posed as:

(7) $$\begin{array}{cc} & \min_{H_{i},t}\;w_{j}{\left(\frac{1}{dF_{\text{in}}/\mathrm{dx}}\right)}^{2}+w_{j+M}\frac{1}{RT_{2}-RT_{1}}\\ & \text{s.t.}\;H_{i}\in G_{M},\;i=1,\text{…},M\\ & \;\;H_{i+1}=H_{i},\;i=1,\text{…},M-1 \end{array}$$

where *M* is the number of metastable units, *RT*_2_ and *RT*_1_ are the resetting time under two different pressure profiles (see Figure 4f; Figure S19a, Supporting Information), *w*_*j*_ are the optimization weights, and *G*_*M*_ is the set that contains the metastable configurations. Notice that the last part of the objective function can be substituted by *w*_*j* + *M*_×Target*RT* − *RT* (where Target*RT* is the target resetting time) to design for a specific reset time of the units under a pressure profile. Despite setting most of the geometric parameters, we included the stiffness term in our objective function to maximize the grasping capacity for our pick‐and‐place application indirectly. The full dynamic response predicted by the model shows three different snap‐through instability and two snap‐backs in the two simulated cases (Figure 4f). As expected, to maximize the resetting time at two different conditions, the algorithm would converge to the highest possible metastable unit (see Table S10, Supporting Information) for the given thickness (see Figure S1b, Supporting Information).

### 2.4 Dome Phalanx Robot Architectures

**Figure 5:**
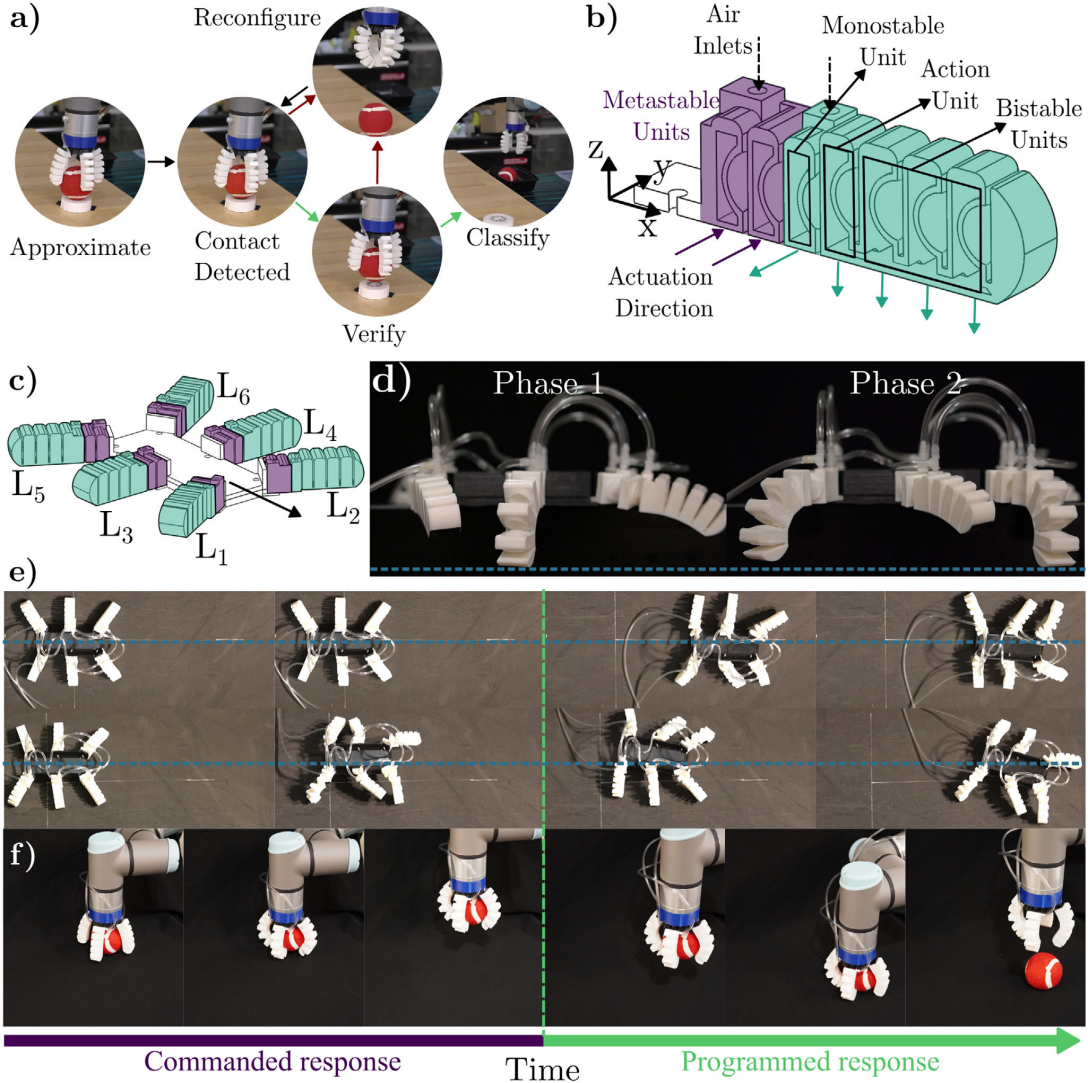
Dome Phalanx Robot (DPR) architectures and applications. a) Classification loop for the DPG. The gripper reconfigures each design's stable state to sort the objects by size and weight (see Movie S7, Supporting Information). b) The DPW actuator has two different zones and three bending directions. The action unit is utilized to create turning at the given pressure command. d) DPW architecture connected to create the tripod gate (*L*_1_‐*L*_4_‐*L*_5_ and *L*_2_‐*L*_3_‐*L*_6_). Forward units are utilized to create turns. e) Oscillating phases of the DPW with the robot at 30 psi of pressure. f) Snapshots of embodied pick‐and‐place tasks: A single pressure input is given for the robot to pick the object (Commanded response) and then release it depending on the viscoelastic material response (Programmed response; see Movie S6, Supporting Information). g) Snapshots of the walking trajectory of the DPW. A command is given by increasing the pressure in the first cycle to enable turning in both directions (Programmed response) (see Movie S9, Supporting Information).

To explore the capabilities of our design approach and the advantages of leveraging multistability in soft robotics, we fabricated two soft robot topologies (Dome Phalanx Robot ‐ DPR). We demonstrate different operation modes using the proposed design methodology and FDM 3D printing to fabricate multiple robot architectures. Specifically, we focus on three types of applications. First, we design a multistable soft gripper with four different stable configurations and a metastable configuration that leverages the viscoelastic response of the material to increase the grasping force. This architecture leverages the embodied states so the gripper can perform object size and weight sorting tasks informed by its pre‐programmed morphology. Second, we realize a hexapod soft robot that walks with alternating tripod gait^[^ ^8^ ^]^ (Dome Phalanx Walker ‐ DPW), by generating horizontal and vertical two‐way bending functions combining bistable and metastable units on different directions (see **Figure** **5**b–d). We impose different actuator geometry and optimization constraints (detailed below) on the model for each operation mode to ensure that the resulting optimized actuators are accurately designed for each specific task. Third, we combine bistable and metastable dome geometries to embody a pick and place robotic behavior into the designed morphology (see Figure 5f; Movie S6, Supporting Information).

**1) Multistable soft robot**: We further leverage the characteristics of our multistable DPF by combining different static and dynamic configurations. We utilized our strategy to design a multistable Dome Phalanx Gripper ‐ DPG with four different stable configurations and a variable dynamic behavior (See Figure S21a, Supporting Information). Given this, four different object sizes are encoded into the structure, which allows for object sorting within the range of the target configurations. A metastable unit is integrated into the tip of each DPF to enhance its classification capabilities and allow the gripper to reconfigure momentarily into a stiffer configuration, allowing it to pick objects of the same size but with different masses. The control loop is closed by combining the morphological information of the robot with a contact sensor in one of the dome units (see Section A.8.1, Supporting Information, for details) to detect when the gripper is perturbed from its design stable position. For this application, the robot base length and DPF angle with respect to the base (see Figure S21, Supporting Information, for reference) are integrated, and four different apertures are programmed into the gripper morphology by minimizing for four discrete configurations (see more detail in Section A.8.1, Supporting Information). Given this, the objective function can be modified as follows:

(8) $$\begin{array}{cc} & \min_{H_{i},t,{\text{Unit}}_{L}^{i},{\text{Unit}}_{s}^{i},\theta_{B},\mathrm{Bas}e_{L}}w_{j}{\left(\text{Object}\;{\text{Size}}_{j}-{\text{Tip}}_{\text{dis y j}}\right)}^{2}+w_{M+N+j+1}{\left(\frac{1}{dF_{\text{in}}/\mathrm{dx}}\right)}^{2}\\ & \text{s.t.}\;H_{i+1}\geq H_{i},\;i=1,\text{…},M-1\\ & \;\;\;H_{i}\in G_{M},\;i=M,\text{…},N\\ & \;\;U_{\text{sep}}^{i+1}=U_{\text{sep}}^{i},\;i=1,\text{…},N\\ & \;\;U_{L}^{i+1}=U_{L}^{i},\;i=1,\text{…},N \end{array}$$

where *N* is the number of targeted stable states, *M* is the number of metastable units, and *j* is the number of test objects (1 ⩽ *j* ⩽ *M* + *N*). By doing this, we can optimize for four target positions and maximum stiffness in the fully activated state (four inverted units) and one metastable unit. It is worth noting that for this case, there is a different constraint for the dome height (i.e., *H*_*i* + 1_ > *H*_*i*_), which allows each of the states to be useful for grasping objects while being accessible in a sequential manner (increasing the input pressure). While the model could be expanded to modify the unit cell's dimensions further and determine the optimal number of units, we fixed the number of units at five to ensure at least one active dome per object size for simplicity. We tested our system by performing an inverse design with four different target positions (see Section A.8.1, Supporting Information, for reference), using the same methodology described previously (Section 2.3). This methodology demonstrates the versatility of our DPF and provides an alternative approach to encoding multiple set points within the finger's body by modulating its unit cell geometry. Using this approach, we embed the classification task directly into the gripper's morphology by leveraging the encoded set points to infer object size. A contact sensor (see Section A.8.1, Supporting Information, for details) is integrated into the first unit of one DPF, enabling the detection of perturbations from the gripper's stable configuration. Based on this, we implement a classification loop (Figure 5a): the gripper approaches the object, evaluates contact sensor feedback, and reconfigures if the measured resistance is below a programmed threshold. Upon contact detection, the object is gently lifted with low pressurization (*P* < 5 psi), and slippage is monitored by measuring the contact sensor a second time. If no slippage occurs, the object is classified into one of four size categories (see Movie S6, Supporting Information). If the lift fails, the gripper reattempts by inverting the metastable unit at the tip, increasing stiffness to handle heavier objects of similar size (see Movie S7, Supporting Information). This methodology enables simultaneous size and weight classification by combining the system's mechanical response with real‐time feedback from a single sensor signal. More importantly, given that the robot's morphology purely drives the classification, there is robustness on the specific task, as it decouples from the actuation pressure.

A key characteristic of our multistable soft robots is their intrinsic capacity to attain a desired shape and exert force without the need from external work, commonly from pressure. This allows our robots to work even after sustaining significant damage, which we induce here by piercing our DPF with several needles (see Movie S7, Supporting Information). Notably, our robot maintains its embodied capability to classify objects even under the presence of leaks (illustrated by the oscillating tufts in Movie S9, Supporting Information). This test demonstrates the robustness of the embodied control from our approach, as well as showing the unique damage tolerance afforded by the robot's multistability.

**2) Hexapod walker**: We demonstrate the versatility of our dome units to design a different topology, a soft robotic leg for a hexapod walker (see Figure 5b,c). The presented walker utilizes an oscillation cycle resulting in a tripod gait, which means that three legs (two on one side and one on the opposite side, i.e., *L*_1_, *L*_5_ and *L*_4_ in Figure 5c) move simultaneously forward, while the other three stay in contact with the ground (see Figure 5d). Each leg is realized by rotating the dome unit into different planes to obtain motion in two planes (see Figure 5b). The leg is divided into two zones (highlighted in purple and green in Figure 5b), which are actuated in an alternating sequence to obtain the desired movement. Specifically, actuation of the purple zone results in forward motion of the leg, while actuation of the green zone pushes the leg onto the ground generating contact (see MovieS9, Supporting Information). More importantly, we leverage metastable units in the purple region to expand the range of the oscillating cycle during phase 1 (see Figure S22e and Movie S9, Supporting Information, for a comparison between metastable and monostable cases), utilizing the resetting time to delay the reversion of the domes while transitioning between actuation phases. Consequently, the delay in the metastable units amplifies the overall movement as the leg touches the ground at a farther point than for a monostable counterpart (see Figure S22e, Supporting Information). Furthermore, the green zone is designed to move in the negative y‐axis (‐y) and z‐axis, allowing the leg to touch the ground and move backward to generate the oscillating cycle shown in Figure S22c (Supporting Information). The green zone contains three different types of units: one monostable unit (which gives motion in ‐y direction), three bistable units that provide the negative z‐axis (‐z) movement and can reconfigure to a second stable state, and an action unit that can be either monostable or metastable. The versatility of our robots' units allows us to have two different types of legs. The first type is for front legs (*L*_1_ and *L*_2_) that feature a metastable action unit to generate asymmetric motion (see Movie S9, Supporting Information)  for steering the walking direction (for details, see Section A.9, Supporting Information). The second type comprises the middle and back legs, where we select the action units to be monostable as no asymmetric motion is needed. For the action unit on the front legs, the resetting time is designed to be larger than the one in the purple region, resulting in a programmed response that only depends on the input pressure. As the pressure increases in one of the phases, the action unit is inverted (Commanded response in Figure 5e) inducing asymmetric locomotion in the system (moving the center of mass of the robot). The asymmetric locomotion results from the cycle time of each legs' actuation phase being lower than the resetting time of the action unit (Programmed response Figure 5e). Note that the robot can recover its forward movement by increasing the pressure on the opposite phase, restoring the symmetry in the system with the two action units inverted. Finally, the bistable units on the green zone can be inverted to morph the robot into an arch‐like shape (see Figure S22b, Supporting Information), that increases its payload capacity as it avoids the friction with the ground. As a result, the morphology of the leg embodies a mechanical logic behavior that allows the DPW to move forward, turn in both directions and reconfigure just by controlling the input pressure (see Movie S9, Supporting Information).

**3) Embodied soft robotic tasks**: We utilize our ability to program our system's dynamic response to obtained desired functional behaviors. By leveraging our dome structures' viscoelastic response and geometry, we can pre‐program the duration for which the structure remains activated before returning to its original state. Combining the meta‐ and bi‐stability of the units enables us to design task planning operations (such as pick‐and‐place; see Movie S6, Supporting Information), where the robot can pick an object by activating all units, move to a specific position, and release the object without additional interventions and with just one pressure input (i.e., eliminating the need for a release command; see Figure 5e). We tested this operation mode by utilizing the architecture designed in Section 2.3 ‐ Dynamic response design, to create a DPG architecture. The obtained results show how an object can be held while all units are inverted, even when the system releases pressure (Figure S20, Supporting Information). However, the object is automatically released from the gripper after the metastable units reset, as desired from the programmed dynamics. Using this approach, we can program a simple pick‐and‐place operation by activating all domes or perform a simple pinch operation as demonstrated in Figure S20 and Movie S6 (Supporting Information). Further flexibility in the attainable behavior of our robots can be obtained by including the actuation loading time, which results in the same DPG morphology adapting its response to pick an object and release it at two different times as demonstrated in Movie S6 (Supporting Information).

## 3 Conclusion

In this study, we introduced a modeling framework and inverse design methodology for soft robotics by discretizing their continuous response using multistable structures, enabling us to embody their control into the robot's morphology. We propose two robotic architectures, the Dome Phalanx Gripper and Dome Phalanx Walker, that leverage mechanical instabilities to discretize the robots' configuration space into desired kinematics, which serve as control set points. Exploiting these characteristics, we embodied diverse static and dynamic responses providing an alternative open‐loop control strategy guided solely by the designed geometry and material response for realizing various robotic tasks that would otherwise require closed‐loop approaches. Implementing an energy‐based modeling approach was central to our methodology, enabling us to predict and program the DPF's behavior and units accurately. Consequently, we obtain a robust framework for modeling the robot's static and dynamic responses with a mechanics‐informed parameter discovery procedure that yields high‐fidelity results consistent with experimental data. Significantly, this methodology extends beyond the DPF geometry and its lattice representation, as the combination of mechanics knowledge (in this case from shell theory), data, and recursive feature elimination can be further exploited for a wide variety of geometries and reduced‐order models. Utilizing the predictive capabilities of our model, we successfully implement the inverse co‐design of the DPFs' morphology and controlled functionality, yielding desired target positions, accessible states, and programmable time responses based on the structure geometry and material viscoelasticity. More importantly, we show that we can perform simple classification tasks by combining the robot morphology and simple feedback. We demonstrate that multistability allows for designing damage‐tolerant inflatable robots by significantly reducing the reliance on careful pressurization to achieve desired shape and dynamics. Furthermore, the versatility of our approach is illustrated by enabling the controlled locomotion and steering of a walker by open‐loop simple pressure modulation. As the robot behavior is transformed into a finite, tractable set of positions, implementing closed‐loop control strategies is simplified by tracking a finite number of states instead of the infinite number inherent to continuous systems. Our results show how these characteristics can be de‐signed to allow for dynamic reconfiguration, grasping force modulation, and embodied logic in the robot morphology without needing closed‐loop control. The obtained findings highlight the potential gains in performance when morphology and functionality are concurrently optimized (i.e., co‐designed), a result only feasible via the simplified dynamics and modeling afforded by our approach. Combining our morphological control strategy with simplified sensing can create a streamlined closed‐loop control, where conventional sensors are only used to verify if the robot is in a specific state, thus reducing the need for constant feedback. By encoding the control within the morphology and response of the system, we embody a form of mechanical intelligence offering a reduction in control complexity and forgoing the need for data‐hungry models and expensive electronics. The resulting reduction in system complexity is far‐reaching and paves the way for more efficient, cost‐accessible, sustainable, and adaptable soft robotics applications.

## 4 Experimental Section

### Unit Cell and DPF Fabrication

The unit cells were 3D printed using fused deposition modeling (FDM) on a Raise3D Pro2 printer, utilizing Ninjatek Ninjaflex TPU filament^[^ ^52^ ^]^ and Ninjatek Cheetha TPU.^[^ ^53^ ^]^ Each finger was post‐processed to correct manufacturing errors and seal the air chambers. For the DPG, a PLA base was printed that secures each of the four fingers (see Movie S5, Supporting Information, for assembly).

### Stiffness Measurement Tests

The fingers were loaded onto a specially designed test rig and tested using a universal testing machine (3345 Instron) equipped with a 100N load cell (see Figure S15a, Supporting Information). The test rig constrained all degrees of freedom at the finger's base, with six different positions equally spaced along 90° to ensure that the initial load was always perpendicular to the tested geometry. The force‐displacement response was measured five times per sample at a constant velocity of 10 mm/s. Raw data were evaluated and plotted using Python 3.12.

### Finite Element Analysis

The unit cells were modeled in Abaqus using linear elastic material properties and a combination of S3R and S4R shell elements. A dynamic implicit quasi‐static approach was utilized to capture the structure's instabilities. Geometric nonlinear analysis (Nlgeom) is used, and snap‐through was triggered using a pressure load on the dome while the edges are pinned. The unit cell was initially modeled in the stress‐free state. Simulations were run for different material thicknesses (*t*) and dome heights (*H*). The full geometry of the DPF was also modeled in Abaqus using linear elastic material properties and C3D10 3D elements. Snap‐through was triggered using a pressure load on every inner wall air chamber. As discussed in the analysis, a fixed boundary condition was applied to the first unit. After dome inversion, different relaxing steps were used to achieve the final stable state (see Section A.2, Supporting Information, for details).

### Robot Actuation

Each DPF was actuated using a pressure input controlled by a ball valve. The results displayed in Movies S2 and S3 (Supporting Information) were produced by applying pressure as slowly as possible to observe the multistable phenomenon.

### Pressure and Displacement Measurement

The dynamic behavior of the DPF was captured in detail using a Photron Fastcam Mini UX100 high‐speed camera and the data was processed using Tracker Video Analysis and Modeling Tool. Input pressure was measured using a HONEYWELL ABPDANN010BG2A3 pressure sensor coupled with an Arduino Uno (see section A.7.1, Supporting Information, for more details). Each experiment was performed 5 times and the average values are reported.

## Conflict of Interest

The authors declare no conflict of interest.

## Supporting information

Supporting Information

Supplemental Video 1

Supplemental Video 2

Supplemental Video 3

Supplemental Video 4

Supplemental Video 5

Supplemental Video 6

Supplemental Video 7

Supplemental Video 8

Supplemental Video 9

## Data Availability

The data that support the findings of this study are available from the corresponding author upon reasonable request.
