# Supplementary material for: Embodying Control in Soft Multistable Robots from Morphofunctional Co‐design
Source: Adv Sci (Weinh). 2025 Jul 30;12(32):e03206. doi: 10.1002/advs.202503206 (PMC12407363; doi:10.1002/advs.202503206)
Supplement: Supplementary file 1 — Supporting Information [file ADVS-12-e03206-s008.pdf]

ADVANCED SCIENCE
Open Access

Supporting Information

for *Adv. Sci.*, DOI 10.1002/advs.202503206

Embodying Control in Soft Multistable Robots from Morphofunctional Co-design

*Juan C. Osorio, Jhonatan S. Rincon, Harith Morgan and Andres F. Arrieta**

A Supplementary Material

A.1 Geometry

A.1.1 Unit Cell

The system's bistable constitutive units are composed of a dome-shaped structure encapsulated by a square chamber that allows for pneumatic actuation and reset by applying positive and negative pressure, respectably (see Figure S1a). Each dome unit can be geometrically tuned to exhibit a bistable behavior (Figure S1c (iii)) [24], pseudo-bistable (metastable - Figure S1c (ii)) behavior where the is a snap-through instability, but the unit returns to its zero energy state [47, 48], and a monostable behavior (Figure S1c (i)). These mechanical responses can be adjusted by modifying the height H and thickness t of the unit, as shown in Figure S1b.

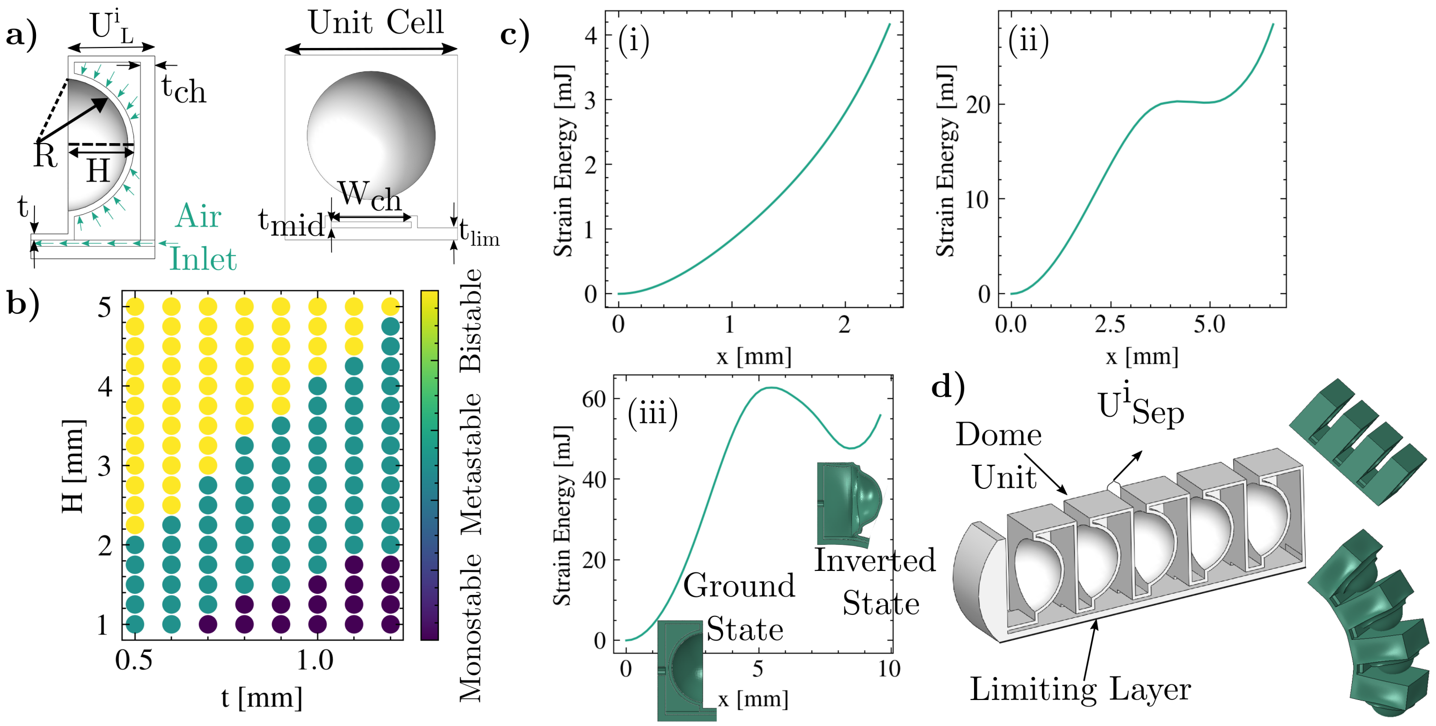

Figure S1: Dome unit geometry and mechanics. a) Unit cell geometry and parameters (Dome unit + air pressure chamber). b) Stability map dependent on dome thickness (t) and dome height (H) for DPF unit cell. c) Monostable (i), Metastable (ii), and Bistable (iii) dome unit behavior. d) Dome Phalanx finger geometry with two possible, stable states (Initial and fully actuated states).

A.1.2 Dome Phalanx Finger (DPF)

The DPF is multistable soft system with sequential dome units (Figure S1d). The finger derives its multistable behavior from domed-shaped shell elements (Figure S1a), and it can be geometrically tuned to reach and retain different final shapes after dome inversion. The DPF performance and stable kinematic configurations can be tuned by changing the dome height (H), Unit Cell size (UC), strain limiting thickness (t_{lim}), air channel dimensions (W_{ch} and t_{mid}), spacing between adjacent cells ($\mathrm{U}^i_{\mathrm{sep}}$), unit cell length ($\mathrm{U}^i_{\mathrm{L}}$), chamber thickness ($t_{ch}$) and dome thickness. Once fully inverted, contact between adjacent units and the strain-limiting layer induces the system's global curvature. The domes on each finger segment support programmable deflections as the domes' final positions dictate the global kinematic configuration (see Figure S2b). The contact between the dome tip and the adjacent unit's chamber provides an additional interaction, contributing to the final curvature after all units are activated and the pressure is removed.
The strain energy stored in the gripper topology in its activated states is the summation of the individual contributions from the inverted domes (see Figure S2c). The magnitude of the interaction between neigh-

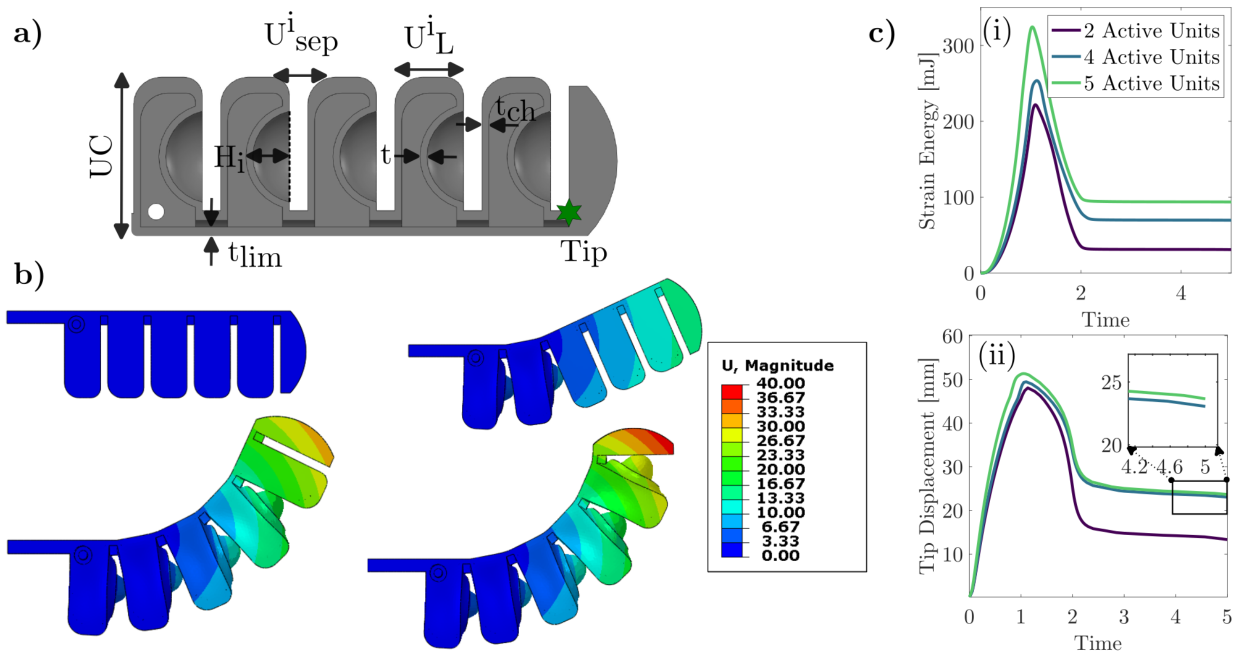

Figure S2: Dome Phalanx Finger geometry and behavior. a) Geometrical parameters that determine the final position and strain energy of the DPF. b) Different stable states of the DPF are encoded by tunning the dome height of each unit cell. c) Strain energy (i) and Tip displacement (ii) as a function of the number of active units. Three different sets of points are encoded into the DPF.

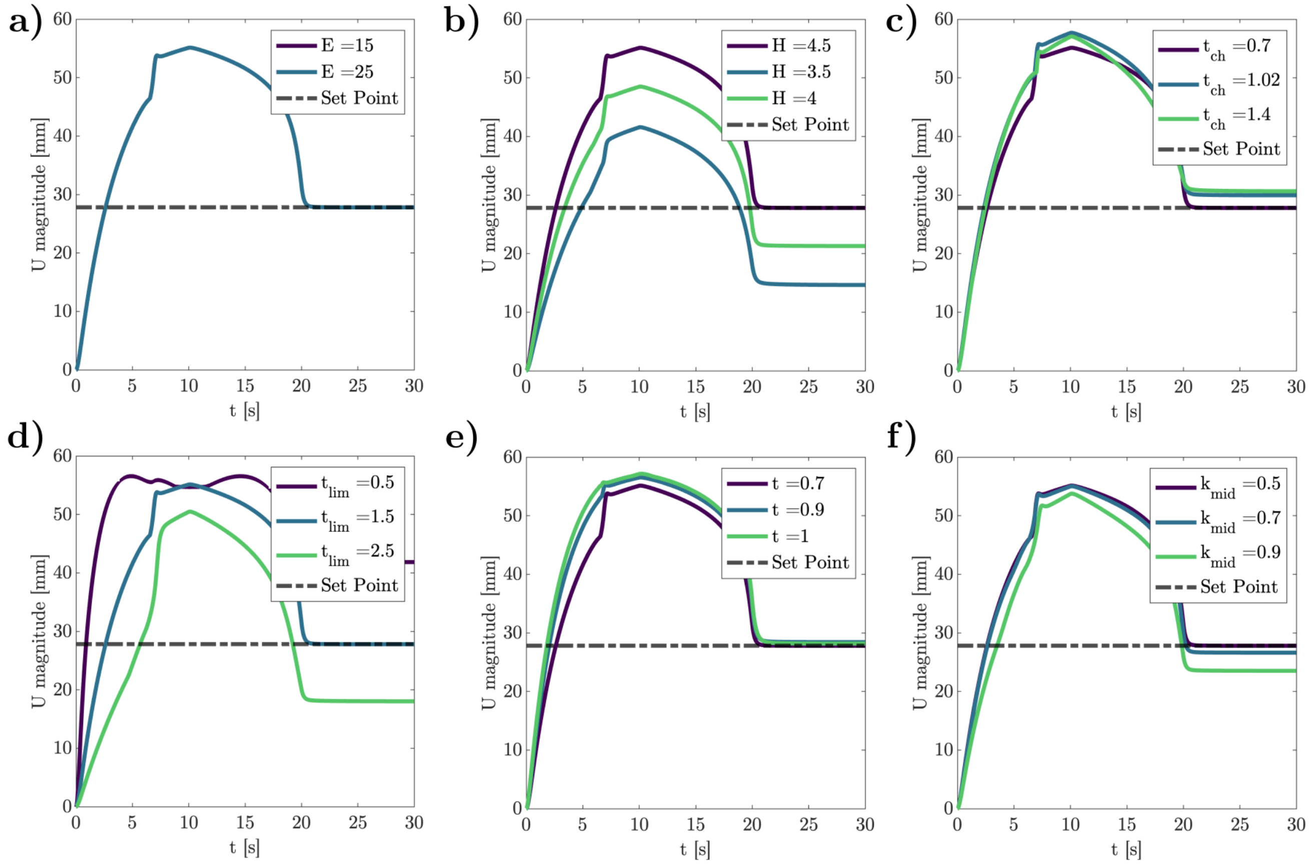

Figure S3: Geometric parameter effect on the time response of DPF. Setpoint, overshoot, and dynamic response can be tuned by combining different parameters. a) Elastic Modulus (E), b) Dome unit Height (H), c) Chamber thickness t_{ch}, d) Limiting layer thickness t_{lim}, e) Dome unit thickness (t), f) Channel with $W_{\mathrm{ch}} = k_{\mathrm{mid}}UC$

boring domes is also affected by dome height (H) and chamber thickness (t_{ch}). Together, dome height and chamber thickness determine the degree of interaction between neighboring segments after dome inversion and yield the system's global curvature (Figure S2b) and the final tip position of the system (Figure S2c). Consequently, various dynamic behaviors can be achieved by adjusting the geometrical parameters, thereby enhancing tunability and control. Specifically, the dome height (H) and thickness (t), along with the limiting layer thickness (t_{lim}), influence the final stable position or set point. Meanwhile, the chamber thickness (t_{ch}) and the air channel dimensions (W_{ch} and t_{mid}) govern the overshoot required to reach a target position (see Figure S3). This tunability enables the gripper's response to be tailored by simply modifying the DPF's geometric characteristics.

A.2 Finite Element Simulations

Finite element simulations are performed using Abaqus and Python scripting to iterate over different geometrical configurations. Given the dominance of the geometric phenomena in the system, all simulations are done using linear elastic material properties.

A.2.1 Unit Cell Simulation

The unit cells are modeled using S3R and S4R shell elements. The mesh is structured to capture the dome symmetry and its appropriate post-buckling behavior. A dynamic implicit quasi-static approach is utilized to capture the structure's instabilities. Geometric nonlinear analysis (Nlgeom) is used, and snap-through is triggered using a displacement control method on the dome while the edges are pinned. The unit cell is initially modeled in the stress-free state. Simulations were run for different material thicknesses (t), dome heights (H), dome radius (R_b), and Elastic Modulus (E). Strain energy vs dome tip displacement is extracted for every step of the simulation (see Figure S4a) which are used to tune the lattice model constant (k_b and α in Equation 1).

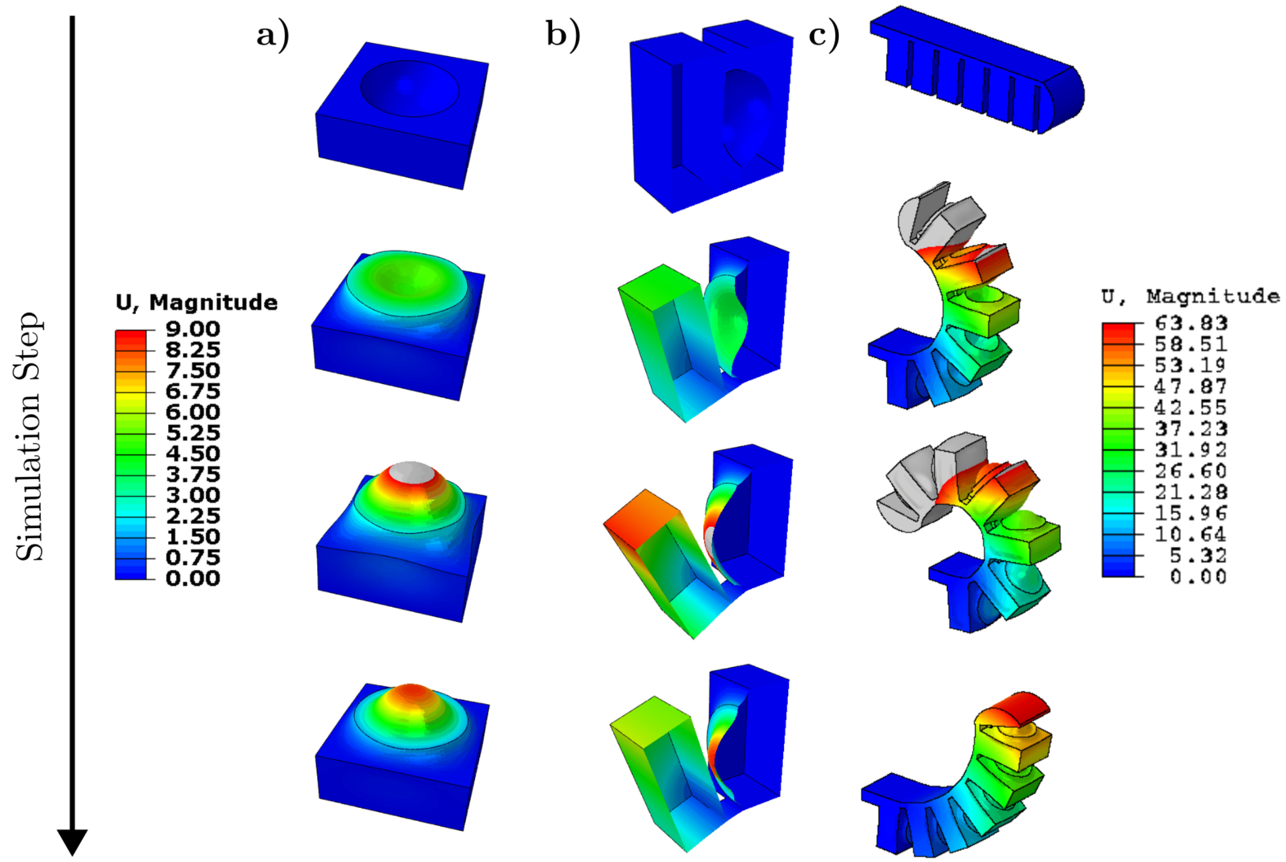

Figure S4: Finite Element (FE) simulations for model parameter tuning and validation. (a) Dome unit inversion sequence used to determine k_b and α. (b) Interaction between dome units to determine d. (c) 3D simulation for validating the model's predictions against FE data.

A.2.2 Dome Unit interaction

The interaction between dome units is modeled by simulating two consecutive units (see Figure S4b). This approach allows us to assess the influence of one unit on the final position of the adjacent dome tip. The interaction is captured using general contact between the surfaces while varying key parameters such as material thickness (t), dome height (H), dome radius (r_b), and elastic modulus (E). Tip displacement is recorded over time, and the final displacement values, as a function of the test parameters, are used to calibrate the parameter d (Equation 1) in the lattice model.

A.2.3 DPF 3D Simulation

The complete geometry of the DPF is modeled using C3D10 3D elements to capture the detailed deformation behavior. Snap-through is initiated by applying a pressure load to each inner wall of the air chambers. 3D simulations are chosen to capture the dynamic behavior under internal pressure loading. A fixed boundary condition is applied to the first unit to prevent rigid body motion. After dome inversion, a series of relaxation steps are performed to allow the structure to reach its final stable state (see Figure S4 c). To validate the lattice model, simulations are conducted for various geometric parameters, including the number of units (N_{Units}), material thickness (t), chamber wall thickness (t_{ch}), elastic modulus (E), dome height (H), and base radius (r_b). The final deformed shape is extracted for comparison with experimental results. The entire DPF geometry and simulation workflow is implemented using Python scripting in Abaqus, ensuring efficient parameter exploration and model validation.

A.3 Energy-based model and constant derivation

A.3.1 Spring Lattice array

Predicting the behavior of multistable DPFs requires consideration of both the unit cells' and the global geometric parameters. By characterizing the contribution of each DPF subcomponent and their interactions, an energy landscape can be built where each minimum corresponds to a stable state of the system. The resulting strain from the energy minimization process dictates the programmed stable shapes with their geometrical and stiffness characteristics. We represent the DPR as a lattice comprising nonlinear [49], linear, and torsional springs (Figure S5a). The springs' stiffness and connectivity allow us to map local extensions and rotations to defined energy contributions. We use nonlinear springs featuring a ground (unstressed) state and an inverted (stressed) to capture the influence of the bistable domes on the system. To appropriately represent the bistable dome behavior, we position the nonlinear springs so that the path of extension coincides with the dome's tip position between the ground and inverted states. Linear springs capture connections between the nonlinear units modeled as struts. In the DPF's case, the strain these segments experience is negligible, behaving as a strain-limiting layer. Consequently, we model these connections as nearly rigid. While the axial strains experienced by this strain-limiting layer are negligible, the layer is sufficiently thick to display bending resistance. We capture the influence of this bending stiffness by introducing torsional springs at the nodes coincident with the strain-limiting layer. The angular displacement experienced by the torsional springs is dictated by the change in the angle ϑ formed by the segments (see Figure S5a).We define $k_l = \frac{Et_{\mathrm{lim}}UC}{U_L}$ and $k_t = \frac{Et_{lim}^3}{12(1-\nu^2)}\frac{UC}{U_{\mathrm{sep}}}$. Where E is the young modulus, ν is the Poisson ratio, and t_{lim} is the thickness of the limiting layer shown in Figure S2a.

Minimizing the system's total energy, $E_{tot} = \sum_{i=1}^{n} E_L + E_{NL} + E_T$ where n is the number of units, constrains the space of possible interactions for the lattice elements into a discrete set of stable states. The rich configuration space of our DPF requires establishing a rational method for providing the initial guess and initializing the minimization process. To this end, we implement a geometric base model to generate initial guesses for the optimization algorithm so that the obtained states are in the neighborhood of physically feasible configurations with improved computational time. The geometric model for the dome phalanx finger assumes a fixed extension of the nonlinear spring and pure rotation from segment to segment. Here, the activated states of the finger (i.e., with units with inverted domes) are determined by extending the length of the linkage corresponding to the bistable dome structure such that it reflects the distance along the central axis between the tip of the dome to the base of the dome chamber. The now extended segment yields an angle ϑ between the present and initial position of the front segment (see Figure S5 a). The model uses this ϑ to calculate the rotation of the subsequent segment, and the process is repeated until the number of segments is exhausted. In this model, the displacement of the overall fingertip can be calculated as a summation of sines and cosines. This purely geometric model does not account for the internal force balancing characteristic of each stable state, which ultimately leads to errors in the configuration predictions. Nevertheless, predictions of a purely geometric model are a powerful tool in finding solutions within the hyperdimensional energy landscape of the spring lattice when we use the outputs of the geometric model to form the initial guess in our search for local extrema. The stable configurations that result from balancing the force and energy contribution of coupled springs differ from the activated configurations determined by the geometric model, but the discrepancy is one easily overcome by the energy minimization process.

A.3.2 Material viscoelastic response

Material time response is captured using a variation of the Kelvin-Voigt viscoelastic constitutive model[54]. The model consists of two components in parallel: a spring (representing the elastic behavior) and a dashpot (representing the viscous behavior) see Figure 3a.

Nonlinear Spring (Elastic Element): The spring represents the material's dome behavior, as explained in the previous section. This spring follows Equation 1.

Dashpot (Viscous Element): The dashpot represents the material's viscous behavior, meaning it resists deformation by dissipating energy as heat. This behavior is modeled by considering two different damping forces, isotropic damping, and internal damping [55]. The isotropic damping for numerical stability on a node i can be written as:

$$F_d^{iso}(\dot{x}_i) = \eta_{iso}\dot{x}_i \tag{S.9}$$

where x_i represents the position and node i and $\dot{x}_i$ represents its velocity. Internal damping is selected to be proportional to the spring coefficients as [55]:

$$F_d^{\text{int}}\,(x_{ij}, \dot{x}_i, \dot{x}_j) = \eta_{\text{int}}\left(\left(1 - \frac{s_{ij}}{\|x_{ij}\|}\right)(\dot{x}_i - \dot{x}_j) + \frac{s_{ij}}{\|x_{ij}\|^3}\left[x_{ij}\cdot(\dot{x}_i - \dot{x}_j)\right]x_{ij}\right) \tag{S.10}$$

where s_{ij} is the rest length of the spring, and $x_{ij} = x_i - x_j$. η_{iso} is selected to guarantee numerical stability, and η_{int} coefficient is obtained by fitting the dynamic behavior to the experiments for different loading conditions. We obtained $\eta = 0.05$ Ton s^{-1} by utilizing the Prony series characterized in [56].

A.3.3 Parameter Tuning

To determine each of our lumped model's parameters, the DPF's fundamental unit cell is isolated into two different parts (see Figure S5a). The dome unit is fitted as a nonlinear spring following Equation 1 and the strain limiting layer is represented by a liner spring to capture stretching and two rotational springs to capture the bending rigidity. Each of the constants for these springs needs to be tuned for different geometrical cases to produce an accurate model for inverse co-design. Given Equation 1, the parameters to be determined for the nonlinear spring are k_b, α, and d. These parameters are functions of the dome unit's mechanical behavior (stiffness and energy barrier) as well as the interaction between the dome and the wall of the air chamber of the subsequent unit cell. The procedure to find each constant as a function of the geometrical parameters of the dome unit cell is as follows. First, different finite element simulations of the unit cell are utilized to determine the best-fitted values for k_b, α, and d (Figure S5b) for different geometrical cases (H and t, same as shown in Figure S1). Different parameters, such as elastic modulus, are examined to establish their influence on the strain energy and tip displacement (Figure S5b) and establish a better understanding of the governing parameters to be considered to represent the nonlinear spring accurately. Equation 1 is fitted to the FE data using the nonlinear least square method to obtain the overall behavior of these parameters in terms of H and t. Results for these fitted parameters can be observed in Figure S5c, and the energy-displacement curve with the fitted parameters can be observed in Figure S6a.
Once the values are obtained, we use Recursive Feature Elimination (RFE) [50] combined ridge regression to determine an expression for k_b, α, and d as functions of the dome height (H), dome thickness (t), and dome curvature (R). By using RFE, we can automatically determine the relevance of each feature in our model. Given the main task of inverse design of multistable actuators, a general and scalable model is needed to cover different design cases. We establish the model by considering three different non-dimensional relations:

$$\pi_1 = \frac{t}{H} \quad \pi_2 = \frac{t}{R} \quad \pi_3 = \frac{H}{R} \tag{S.11}$$

where π_1 represents the load-carrying capacity of the shell, π_2 is the curvature-to-thickness ratio, and π_3 quantifies the dome shallowness. Using these three non-dimensional quantities, the following model is fitted:

$$\alpha = \mathbf{C}(\pi_1, \pi_2, \pi_3)\xi_\alpha \quad \mathbf{k_b} = \mathbf{C}(\pi_1, \pi_2, \pi_3)\xi_k \quad \mathbf{d} = \mathbf{C}(\pi_1, \pi_2, \pi_3)\xi_d \tag{S.12}$$

$\mathbf{C}(\pi_1, \pi_2, \pi_3)$ is a matrix of all the possible candidates and interactions of the non-dimensional relations. The $N \times M$ matrix can be written as:

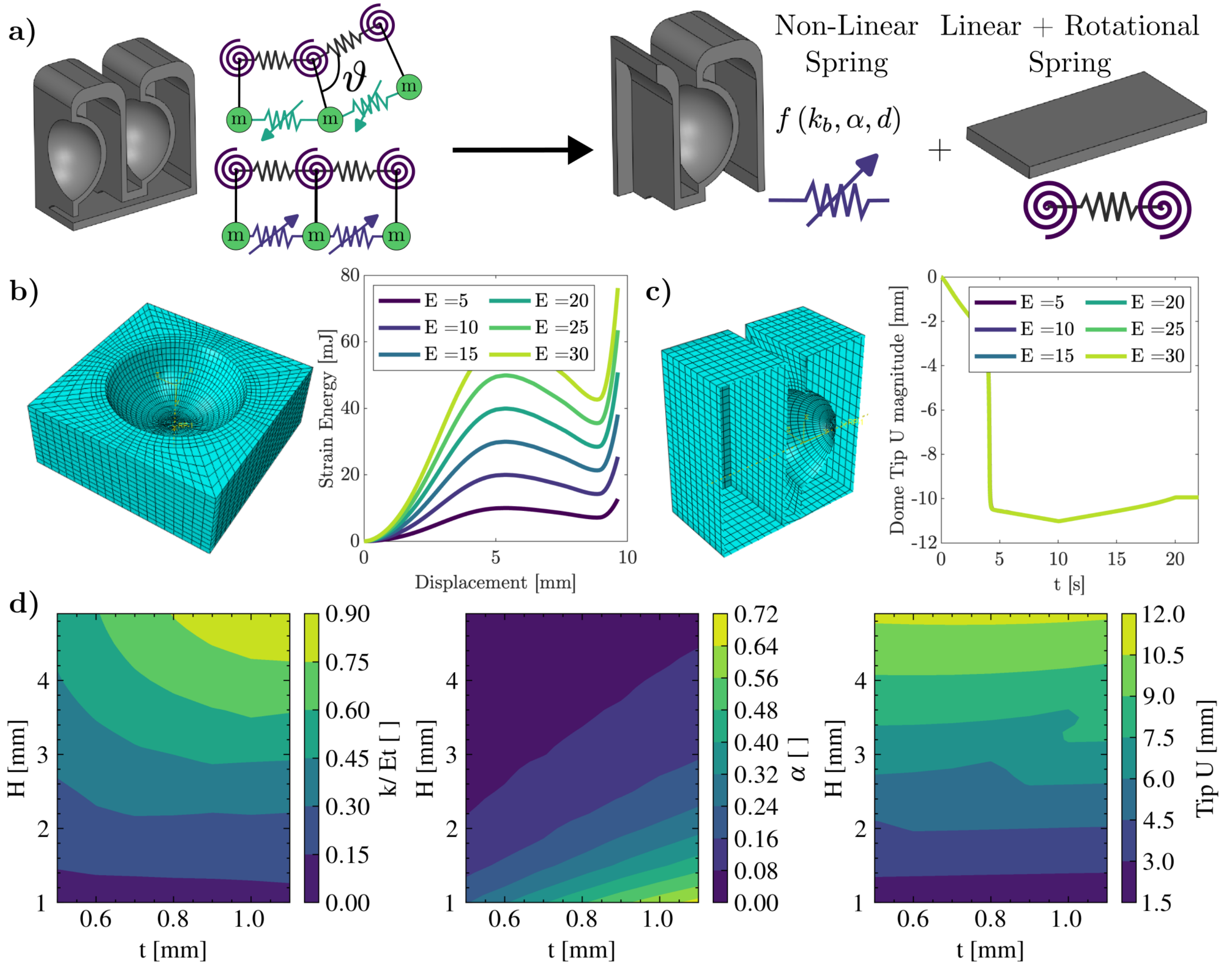

Figure S5: a) DPF fundamental unit cell isolated into different lumped parameter spring elements. (nonlinear + linear + rotational springs) b) nonlinear spring parameter behavior over different dome heights H and dome thickness t.

$$\mathbf{C}(\pi_1, \pi_2, \pi_3) = \begin{bmatrix} \pi_i & \pi_i^2 & \dots & \pi_i^n & \pi_i\pi_j & \dots & \pi_i^n\pi_j^m \end{bmatrix} \tag{S.13}$$

where superscripts n and m represent the maximum degree of the polynomial use for the regression and subscripts i and j represent each of the non-dimensional numbers. For the parameter α we can fully represent this regression as:

$$\begin{bmatrix} \alpha(H_1, t_1, R_1) \\ \alpha_2(H_2, t_2, R_2) \\ \vdots \\ \alpha_k(H_k, t_k, R_k) \end{bmatrix} = \begin{bmatrix} \pi_i(H_1, t_1, R_1) & \dots & \pi_i^n(H_1, t_1, R_1) & \pi_i\pi_j(H_1, t_1, R_1) & \dots & \pi_i^n\pi_j^m(H_1, t_1, R_1) \\ \pi_i(H_2, t_2, R_2) & \dots & \pi_i^n(H_2, t_2, R_2) & \pi_i\pi_j(H_2, t_2, R_2) & \dots & \pi_i^n\pi_j^m(H_2, t_2, R_2) \\ \vdots & & & & & \\ \pi_i(H_k, t_k, R_k) & \dots & \pi_i^n(H_k, t_k, R_k) & \pi_i\pi_j(H_k, t_k, R_k) & \dots & \pi_i^n\pi_j^m(H_k, t_k, R_k) \end{bmatrix} \begin{bmatrix} \xi_1 \\ \xi_2 \\ \vdots \\ \xi_k \end{bmatrix} \tag{S.14}$$

Note that we assume that the library of terms used to obtain $\mathbf{C}(\pi_1, \pi_2, \pi_3)$ has a sufficiently rich column

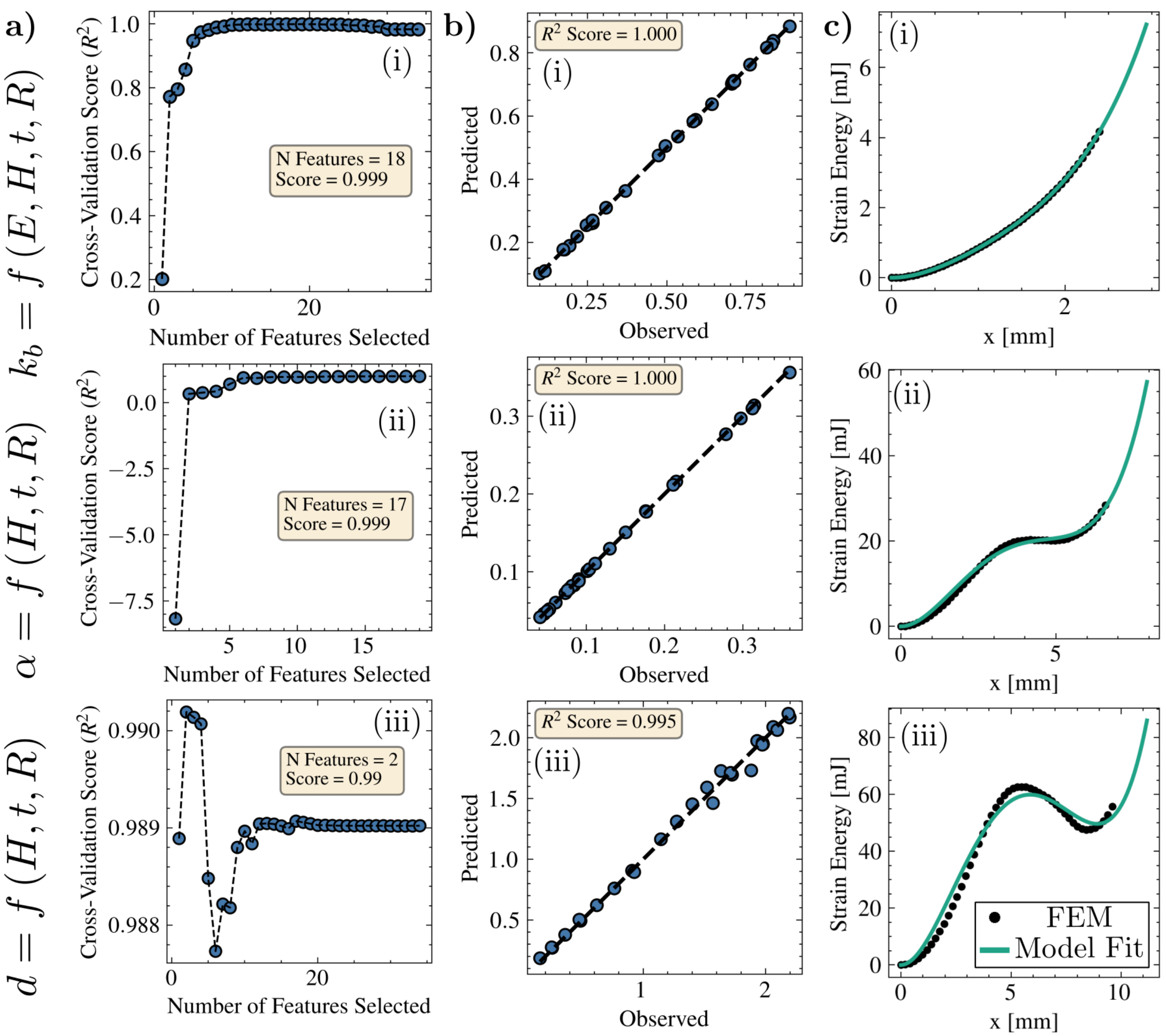

Figure S6: Parameter-fitting results for α, k_b, d as a function of geometric parameters. a) RFE with ridge regression cross-validation vs the number of features (training data) for k_b (i), α (ii), and d (iii); b) Prediction vs observation plot on test data for the reduced model for k_b (i), α (ii), and d (iii); c) Model fit for energy as a function of geometric parameters for monostable (i), metastable (ii) and bistable (iii) cases.

space that the behavior will be represented by Equation S.12, and it can be written as a linear combination of the weights ξ. The data is split into 70% train and 30% test data sets to calculate the accuracy of our model. Observation vs prediction plot and estimation error for the three different variables can be observed in Figure S6b (ii), where a good fit between the proposed model and the test data is observed. Moreover, we use RFE with cross-validation (cv) on the test data to eliminate features and retest the model to determine its accuracy. R^2 for the cross-validation data as a function of the number of features can be observed in Figure S6b(i). This gives a reduced model with similar prediction capabilities and the most relevant geometric relations of the problem, and yields an accurate representation of the behavior (see Figure S6b (iii)).
The final expressions for each of the nonlinear spring parameters can be written as:

$$k_b = f\left(E, \pi_1, \pi_2, \pi_3\right) = \frac{E}{R^2}\left(-\frac{1.97Rt^5}{H^3} + \frac{7.4H^2t^3}{R^2} + \frac{3.5Rt^4}{H^2} + \frac{0.37H^2t^2}{R} + \frac{42.2t^5}{H^2} - \frac{35.8Ht^4}{R^2} + \frac{71.8t^5}{HR} - \frac{3.4Rt^3}{H} - \frac{67.7t^4}{H} + 4.2Rt^2 + 11.1t^3\right) \tag{S.15}$$

$$\alpha = f\left(\pi_1, \pi_2, \pi_3\right) = \frac{0.4H^3}{R^3} - \frac{0.6t^3}{H^3} - \frac{2.9H^2t}{R^3} - \frac{0.9H^2}{R^2} - \frac{9.0t^3}{H^2R} + \frac{1.5t^2}{H^2} - \frac{3.9t^3}{HR^2} + \frac{9.2Ht}{R^2} + \frac{17.4t^2}{HR} + \frac{0.6H}{R} + \frac{17.8t^3}{R^3} - \frac{19.5t^2}{R^2} - \frac{6.1t}{R}. \tag{S.16}$$

$$d = f\left(\pi_1, \pi_2, \pi_3\right) = 2.14H + 0.25t - H - \mathrm{U_{sep}} - t_{ch}/2 - t/2 \tag{S.17}$$

where H, t and R are dome unit parameters, E is the elastic modulus of the material,$\mathrm{U_{sep}}$ and t_{ch} is the separation between units (see Figure S2).

A.3.4 Derivation of Jacobian terms

Here we detail the derivation for the energy terms of the linear. non-linear and rotational springs.

Linear Springs: The force on a node i at position x_i interacting with and adjacent node j at a position x_j is given by:

$$F_L(x_i, x_j) = \nabla_{x_i} E_L = \frac{1}{2} k_l \nabla_{x_i} \left(||x_{ij}|| - s_{ij}\right)^2 = k_l \left(\frac{s_{ij}}{||x_{ij}||} - 1\right) x_{ij} \tag{S.18}$$

where $x_{ij} = x_i - x_j$ and s_{ij} is the rest length of the spring.

Nonlinear Springs: The term term of adjacent nodes i and j connected by a bistable spring can be written as:

$$E_{NL} = \frac{1}{2} k_b \bar{x}^2 \left(1 + (1-\alpha)\left(\frac{\bar{x}^2}{d^2} - 2\frac{\bar{x}}{d}\right)\right) \tag{S.19}$$

where $\bar{x} = ||x_{ij}|| - s_{ij}$. Given this, the non-linear spring forces can be written as:

$$F_{NL}(x_i, x_j) = \nabla_{x_i} E_{NL} = k_b \bar{x} \left(1 + (1-\alpha)\left(2\frac{\bar{x}^2}{d^2} - 3\frac{\bar{x}}{d}\right)\right) \frac{x_{ij}}{||x_{ij}||} \tag{S.20}$$

Rotational Springs: The force on a node i due to bending at the limiting layer is given by

$$F_T = \nabla_{x_i} E_T(\vartheta) = k_\vartheta \left(\vartheta - \vartheta_0\right) \nabla_{x_i} \vartheta \tag{S.21}$$

This force is an interaction between three nodes, thus the gradient ∇_{x_i} is computed with respect to $x_i \in x_0, x_1, x_2$. The relevant vectors can be written as

$$e_0 = x_1 - x_0 \tag{S.22}$$

$$e_1 = x_2 - x_0 \tag{S.23}$$

with $\hat{e}_i = e_i/||e_i||$ denotating the normal vector. We can calculate the gradient of ϑ as

$$\nabla_{x_i} \vartheta = \nabla_{x_i} \left(\cos^{-1}\left(\hat{e}_0 \cdot \hat{e}_1\right)\right) = -\frac{\nabla_{x_i}\left(\hat{e}_0 \cdot \hat{e}_1\right)}{\sin \vartheta} \tag{S.24}$$

The gradients with respect to each component of x_i may be expressed as

$$\nabla_{x_0}\left(\hat{e}_0\cdot\hat{e}_1\right) = -\frac{e_0+e_1}{||e_0||||e_1||} + (e_0\cdot e_1)\left(\frac{e_0}{||e_0||^3||e_1||} + \frac{e_1}{||e_0||||e_1||^3}\right) \tag{S.25}$$

$$\nabla_{x_1}\left(\hat{e}_0\cdot\hat{e}_1\right) = \frac{1}{||e_0||||e_1||}\left(e_1 - \frac{e_0\cdot e_1}{||e_0||^2}e_0\right) \tag{S.26}$$

$$\nabla_{x_2}\left(\hat{e}_0\cdot\hat{e}_1\right) = \frac{1}{||e_0||||e_1||}\left(e_0 - \frac{e_0\cdot e_1}{||e_1||^2}e_1\right) \tag{S.27}$$

Using this expression, we can calculate $\nabla\left(E_L(x_{ij}) + E_{NL}(x_{ij}) + E_T(x_{ij})\right) = F_L + F_{NL} + F_T$.

A.4 DPF Static and Dynamic Response

Using the parameters from the fitting process, we can predict stable state configurations for the DPF with the resulting model. Given that the nonlinear elements of the gripper are independently stable, the expected number of states for a given dome phalanx finger topology follows 2^n, where n is the number of bistable segments on the finger. The model can determine the behavior of a given design in orders of magnitude less time than the FE numerical simulations, which makes it feasible for iterating through potential configurations and optimizing for the best design according to a given task (e.g., position and grasping force).

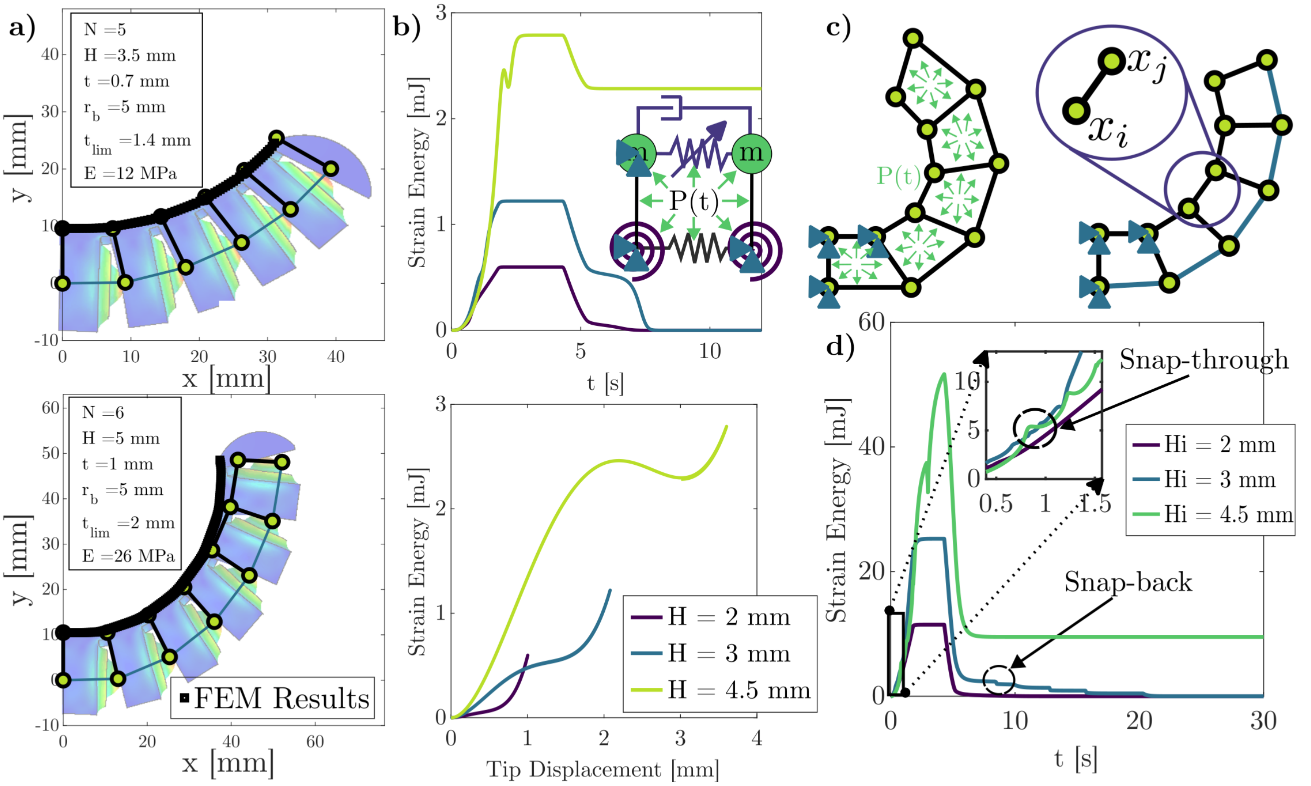

Figure S7: Static and dynamic response of the DPF. a) Stable states are found by minimizing the strain energy. A comparison with FE simulations for five domes of the same height. b) Dynamic response of lattice model with viscoelastic dampers c) Schematic representation of applied load and final DPF position. d) Five-segment DPF dynamic response for different dome H.

Static response: The static response of the finger is predicted by minimizing the total energy of the geometry. We performed this process using Matlab's fmincon sqp algorithm [57]. Our algorithm can predict different final shapes depending on the initial guess geometry. Results for the model are compared with FE simulations, where various points of the limiting layer are compared (see Figure S7a). Results show that our model can predict the final shape of the DPF for different geometric parameters with an error below 3% compared with FE simulations (see Table S1 for an extended comparison).

Dynamic response: TThe model can be further expanded by including point masses on each of the segments to represent the total mass of the system and dampers to capture the viscoelastic response of the material. By implementing these elements and determining its Jacobian terms (see section A.3.4), a dynamic model can be constructed to capture the time response of the DPF. The equations of motion for the system can be written as

$$[M]\ddot{x}_i + F_d^{int}(x_{ij}, \dot{x}_i, \dot{x}_j) + F_d^{iso}(\dot{x}_i, \dot{x}_j) + \nabla\left(E_L(x_{ij}) + E_{NL}(x_{ij}) + E_T(x_{ij})\right) = F_{ext}(x_{ij}, t) \quad \text{(S.28)}$$

where $[M]$ is the mass matrix of the system, $F_d^{int}(x_{ij}, \dot{x}_i, \dot{x}_j)$ is the internal damping, which can be modeled as a dashpot in parallel to each spring (see Figure 3a) and $F_d^{iso}(\dot{x}_i)$ is the isotropic drag, which acts as an

external force on each node proportional to velocity ($\dot{x}_i$) [55]. $F_{ext}(x_{i,j}, t)$ is the external actuation pressure that can be calculated as $F_{ext}(x_{ij}, t) = A(x_{ij})P(t)$. Where $A(x_{ij},)$ is the area of the face where the pressure is applied. By substituting $\dot{x}_1 = x_2$ and $\dot{x}_2 = \ddot{x}_i$, we can rewrite the system as

$$\begin{bmatrix} \dot{x}_1 \\ \dot{x}_2 \end{bmatrix} = \begin{bmatrix} x_2 \\ -[M]^{-1}\left(F_d^{int}(x_{ij}, \dot{x}_i, \dot{x}_j) + F_d^{iso}(\dot{x}_i, \dot{x}_j) + \nabla\left(E_L(x_{ij}) + E_{NL}(x_{ij}) + E_T(x_{ij})\right) - F_{ext}(x_{ij}, t)\right) \end{bmatrix} \tag{S.29}$$

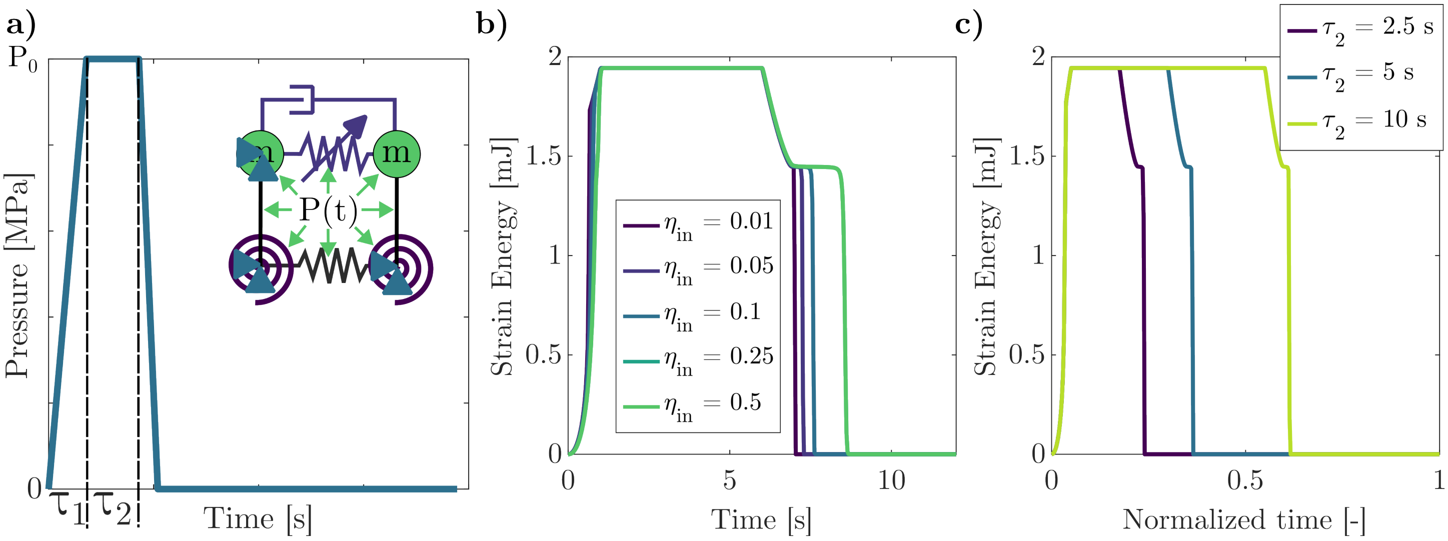

Figure S8: Lattice model dynamic response for dome unit cell. a) Internal pressure over time $P(t)$. Loading time τ_1 and applied load time τ_2. b) Effect of internal damping on dynamic response. c) Effect of applied load time (τ_2) on metastable unit dome reseating time.

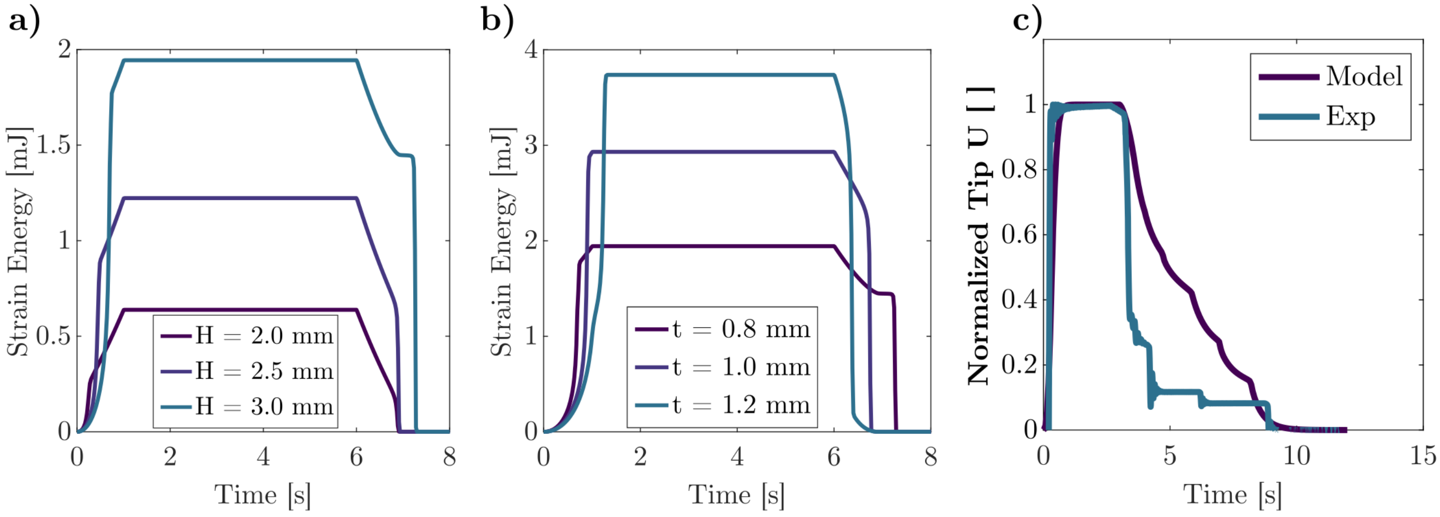

Figure S9: Metastable dome unit analysis. a) Effect of dome height in resetting time of the units (t = 0.8 mm). b) Effect of dome thickness in resetting time of the units (H = 3.0 mm). c) Comparison between experimentally recorded data shown in Figure 2c and our proposed model.

The system is solved using the Runge-Kutta time integration method for loading and unloading external force conditions. We explore the dynamic behavior of a five-segment DPF by applying the internal pressure as shown in Figure S7c, where we obtain the final stable state as shown and the dynamic response shown in Figure S7d. As expected, the model can capture the dynamic response of a bistable, metastable, and monostable DPF, where snap-through and snap-back phenomena are observed. Furthermore, we analyze the temporal response of the metastable units to assess how geometrical parameters and material damping

influence the resetting time. Our results show that adjusting the dome unit's thickness modifies the resetting time (see Figure S9b). However, an even more pronounced effect is observed when varying the loading duration (see Figure S8c). This suggests that after manufacturing the DPF, additional tunability can be achieved by controlling the applied pressure (see Movie 6). The resetting time response is validated against experimental data to assess its predictive accuracy. As shown in Figure S9c, the model effectively captures the time scales of a fully metastable DPF. However, due to the inherent randomness in the resetting process when all units are metastable, the tip displacement is not precisely predicted. Nevertheless, this tool enables the design of both the static and dynamic responses by programming the resetting time scales of the metastable domes (see Movie 2 for experimental comparison).

A.5 Model Validation and Simulation time

The lattice model is evaluated under different geometric configurations as shown in Table S1.

	N Segments	R_b	H/R_b	t	E	t_{lim}	Error %
1	5	5	0.7	0.7	5	1.4	1.37
2	5	5	0.8	0.8	5	1.6	0.62
3	5	5	1	1	26	2	0.51
4	5	8	0.7	0.75	5	1.5	0.51
5	5	8	0.8	1	26	1.6	2.73
6	6	5	0.7	0.7	5	1.4	1.42
7	6	5	0.8	0.8	5	1.6	0.6
8	6	5	1	1	26	2	0.4
9	6	8	0.7	0.75	5	1.5	0.44
10	6	8	0.8	1	26	1.6	2.47

Table S1: List of validation cases for spring model. Error % reference to the difference between model and FE analysis.

Different numbers of segments, elastic modulus, and limiting layer thickness are included to demonstrate the versatility of our approach. The error between the model and the simulation is calculated by comparing the difference between the final position of the limiting layer after all units are activated (see Fig S10).

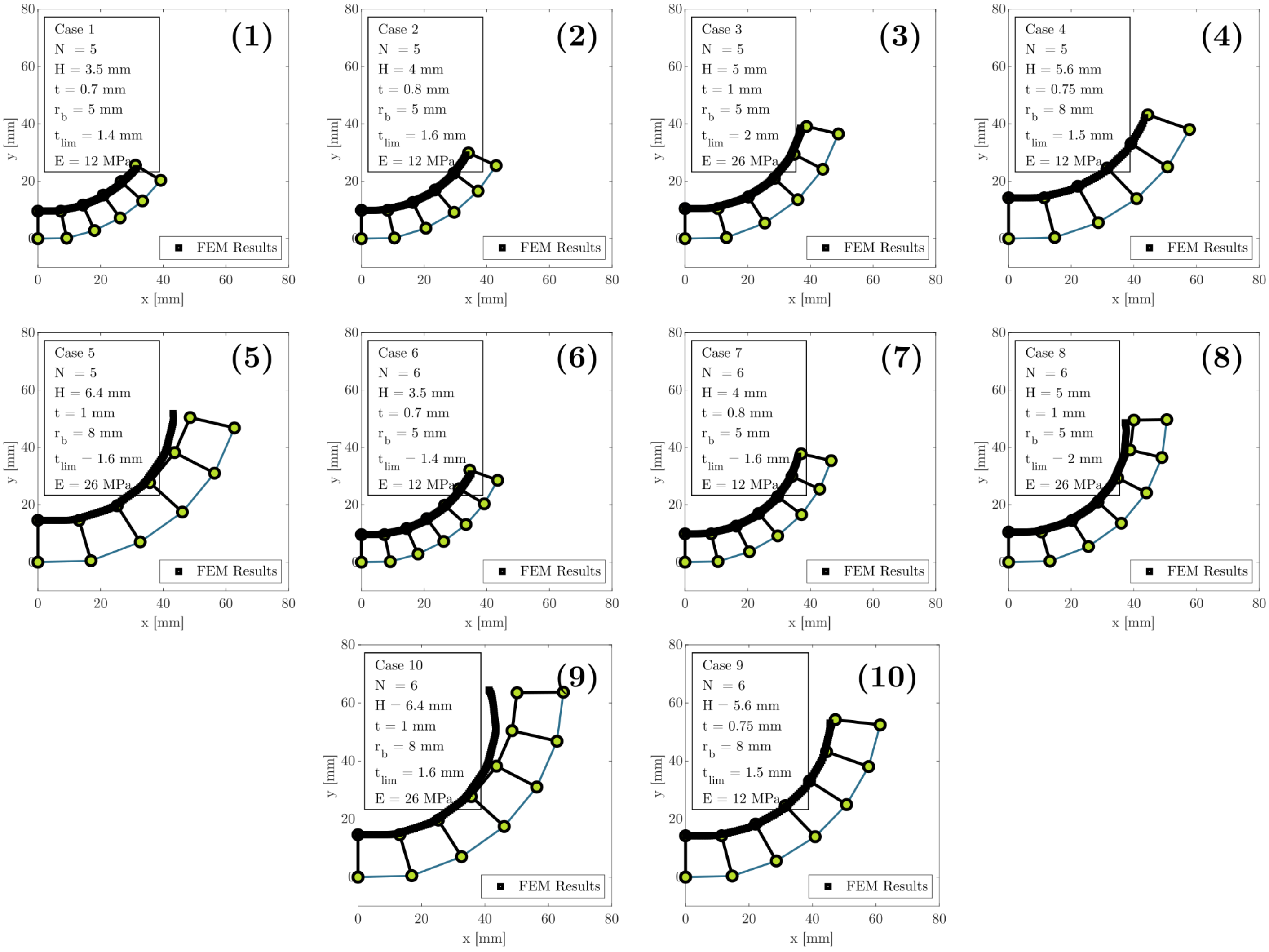

Figure S10: Model comparison with FE method. (1)-(10) Case numbers parameters and error can be observed in Table S1.

Moreover, our model computational cost is evaluated by comparing the total simulation time simulation

time with FE simulations. Results show that the model can yield the final shape of the DPF in less than 1 s. More importantly, the model does not exhibit a coupling between the number of segments and computational time. (see Table S2).

N segments	FE Time [s]	FE Time [h]	Model Time [s]
2	3094	0.86	0.66
3	5960	1.66	0.58
4	12072	3.35	0.64
5	15859	4.41	0.76
6	21455	5.96	0.67

Table S2: Simulation time comparison between FE analysis and spring model.

A.6 Geometry Optimization

A.6.1 Design space for inverse design

We explored the design space of our model by calculating the tip displacement, curvature, and stiffness as a function of the input parameters. Given the width range of parameters, we utilize a Latin Hypercube sampling technique to uniformly sample all of the parameter space (H_i, U^i_{sep}, U^i_L, t and t_{lim}) of the DPF to observe its response after all the units are activated. 1500 samples are taken to guarantee the full exploration of the design space. Analysis for different numbers of units (2 - 7 units) is performed, and the design variables are kept in the ranges listed in Table S3.

	min	max
H_i	3.0	5.0
U^i_{sep}	1.0	5.0
U^i_L	5.5	10.0
t	0.5	1.0
t_{lim}	1.0	2.0

Table S3: Optimization variables ranges.

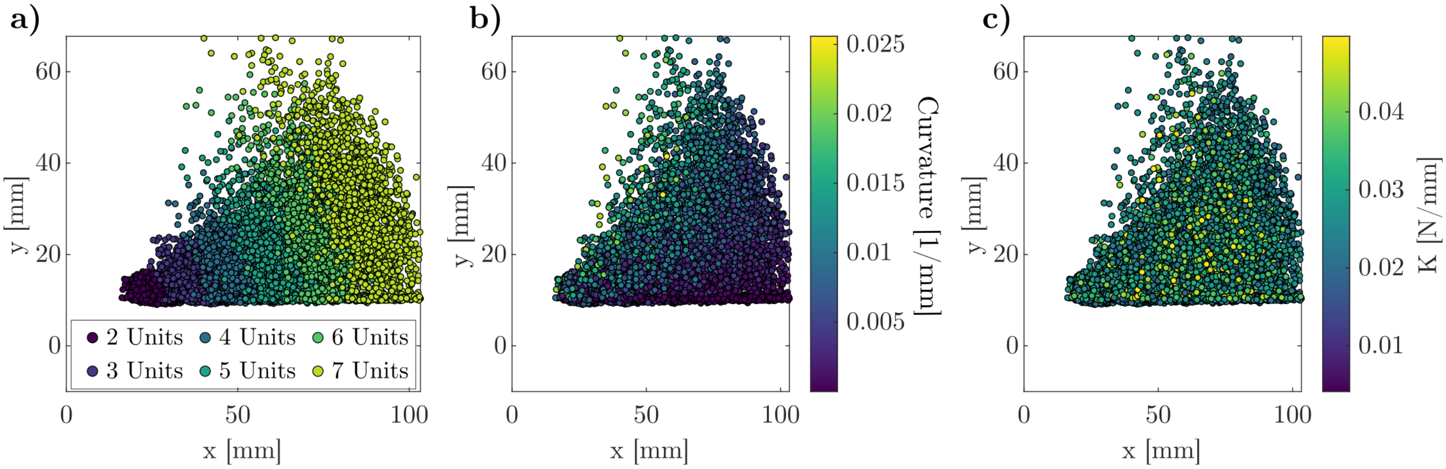

Figure S11: Exploration of the design space. We use our spring lattice model to characterize the deformation modes of structures made of different numbers of units with varying input parameters (Distributed uniformly). a) Tip position design space. b) Curvature design space. c) Stiffness design space.

A.6.2 Inverse problem → Position Optimization

Given a target tip coordinate [x,y] (Target_{xy}), the inverse problem objective function can be written as:

$$\begin{aligned} \min_{H_i,t,U^i_L,U^i_{sep}} \quad & \left(\text{Target}_{xy} - \text{Tip}_{\text{dis}}\right)^2 \\ \text{s.t.} \quad & H_{i+1} \leq H_i,, \quad i = 1,\dots,N-1 \\ & U^{i+1}_{\text{sep}} = U^i_{\text{sep}}, \quad i = 1,\dots,N \\ & U^{i+1}_{\text{L}} = U^i_{\text{L}}, \quad i = 1,\dots,N \end{aligned} \tag{S.30}$$

The final results for the position optimization can be observed in Figure S12, where the five target positions and the final shape predicted by the model are shown. The final geometry and objective function of the optimization algorithm are summarized in Table S4 and S5, where we can observe that for targets 1,2 and 5, the desired position is attained with almost perfect precision.

Table S4: Inverse design problem results for five different targeted positions (NinjaFlex 85A).

	**Target 1** [46 32]	**Target 2** [25 20]	**Target 3** [25 40]	**Target 4** [25 30]	**Target 5** [35 45]
H1	4.5	4.1	4.8	4.5	4.35
H2	4.7	4.3	4.8	4.7	4.35
H3	4.8	4.9	4.9	5.0	4.72
H4	5.0		4.9	5.0	4.87
H5			5.0		4.95
Unit S	2.0	1.0	1.0	1.0	1.01
Unit L	9.9	7.0	7.3	7.0	8.99
t	0.6	0.62	0.68	0.62	0.75
t_{lim}	1.5	1.3	1.2	1.4	1.25
UC	15.0	15.0	15.0	15.0	15.0
K_{dis}	0.009	0.025	0.01	0.018	0.007
**Obj**	0.2	0.2	2.9	0.8	0.4

Table S5: Inverse design problem results for five different targeted positions (Cheetah 95A).

	**Target 1** [46 32]	**Target 2** [25 20]	**Target 3** [25 40]	**Target 4** [25 30]	**Target 5** [35 45]
H1	4.87	4.52	4.89	4.96	4.9
H2	4.8	3.93	4.72	4.52	4.87
H3	4.67	3.63	4.52	4.08	4.72
H4	3.98		4.33	3.99	4.35
H5			4.02		4.3
Unit S	2.31	1.10	1.17	1.08	1.52
Unit L	9.83	7.28	6.95	6.93	8.8
t	0.79	0.83	0.65	0.76	0.63
t_{lim}	1.63	1.59	1.19	1.07	1.72
UC	15.0	15.0	15.0	15.0	15.0
K_{dis}	0.093	0.341	0.079	0.112	0.063
**Obj**	0.775	0.056	0.55	0.22	0.32

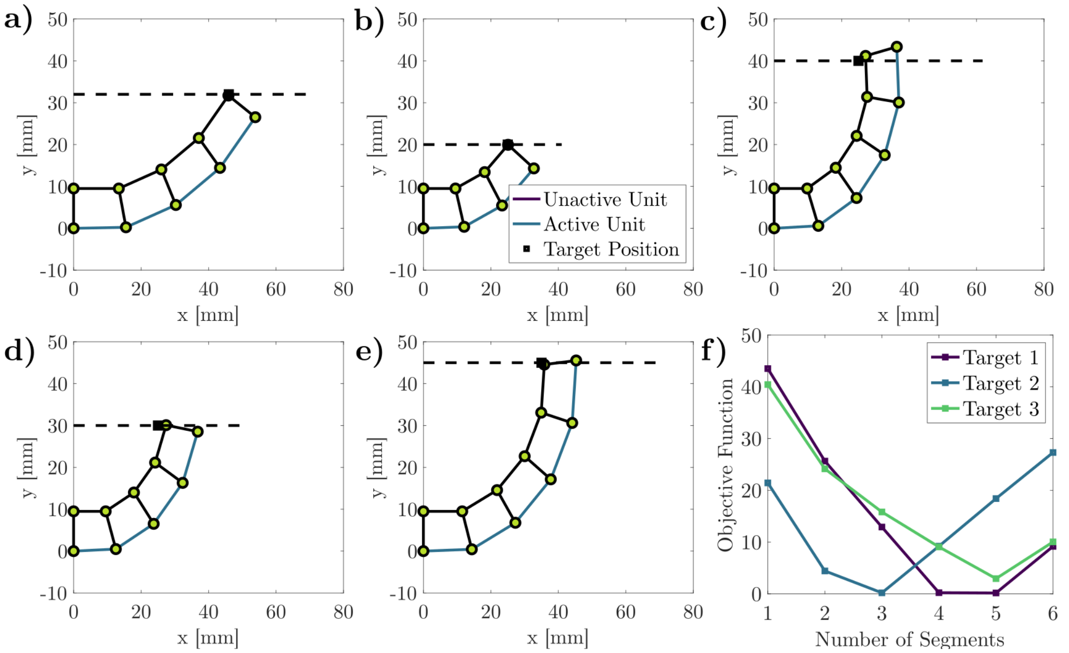

Figure S12: Final results for inverse design problem for tip position as an objective. a) Target 1, b) Target 2, c) Target 3, d) Target 4, e) Target 5, f) Objective function vs Number of segments for the first three target positions.

A.6.3 Inverse problem → Position + Stiffness Optimization

We further expand our model to include stiffness optimization in our objective function. The finger's stiffness is calculated by using the dynamic model described in section A.4, by perturbing the structure from a programmed stable configuration (i.e., set point). We determine the system's total internal force by solving the dynamic equation:

$$[M]\ddot{x}_i + F_d^{int}(x_{ij}, \dot{x}_i, \dot{x}_j) + F_d^{iso}(\dot{x}_i, \dot{x}_j) + F_{\text{in}} = F_{ext}(x_{i,j}, t) \quad \text{(S.31)}$$
$$F_{\text{in}} = \nabla\left(E_L(x_{ij}) + E_{NL}(x_{ij}) + E_T(x_{ij})\right)$$

where $F_{\text{ext}}(x_{i,j}, t)$ is a follower force applied at the tip of the DPF (see Figure 4d) to perturb the DPF from its equilibrium position. The gradient of the internal forces in the structure, F_{in}, is obtained through the analytical expression described in Section 2.2. The force is applied at a constant load rate of 5 mm/min, ensuring a quasi-static regime. The stiffness of the DPF in the desired state is calculated by determining the internal force (F_{in}) and the corresponding tip displacement over time (see Figure 4). A linear fit is then applied to the resulting data. Using this methodology, the DPF's geometry can be optimized to achieve a specific target position with maximum stiffness in the vicinity of that stable state (see Figure 4e).
By considering the finger stiffness during the design, the grasping force is increased by changing the geometric parameters. Results for the optimization algorithm, where the same number of segments as in Table S4 and S5 is maintained, are shown in Table S6 and S7.

Table S6: Inverse design problem results for five different targeted positions with maximum stiffness (NinjaFlex 85A).

	**Target 1** [46 32]	**Target 2** [25 20]	**Target 3** [25 40]	**Target 4** [25 30]	**Target 5** [35 45]
H1	4.63	4.10	4.74	4.81	4.41
H2	4.67	4.27	4.83	4.82	4.58
H3	4.90	4.49	4.85	4.87	4.71
H4	4.98		4.86	4.92	4.81
H5			4.97		4.92
Unit S	2.04	1.00	1.08	1.02	1.07
Unit L	9.94	7.00	7.07	7.03	8.85
t	0.83	0.86	0.81	0.85	0.83
t_{lim}	1.5	1.36	1.45	1.35	1.4
UC	15.0	15.0	15.0	15.0	15.0
K_{st}	0.011	0.0283	0.0146	0.0204	0.009
Improvement	1.22	1.13	1.46	1.13	1.3

Table S7: Inverse design problem results for five different targeted positions with maximum stiffness (Cheetah 95A).

	**Target 1** [46 32]	**Target 2** [25 20]	**Target 3** [25 40]	**Target 4** [25 30]	**Target 5** [35 45]
H1	4.99	4.66	4.96	5.0	4.9
H2	4.88	4.41	4.9	4.78	4.687
H3	4.86	4.31	4.72	4.69	4.57
H4	4.15		4.4	4.66	4.225
H5			3.92		4.2
Unit S	2.26	1.4	1.11	1.01	1.03
Unit L	9.98	6.85	7.11	6.89	8.92
t	0.9	0.91	0.88	0.99	0.99
t_{lim}	1.89	1.95	1.24	1.66	1.94
UC	15.0	15.0	15.0	15.0	15.0
K_{st}	0.093	0.341	0.079	0.112	0.063
Improvement	1.51	1.61	1.48	2.7	2.37

A.7 Experimental Test

A.7.1 Pressure and displacements Measurements

As shown in Figure S13, an air compressor (Pump DOA-P704-AA) is used to supply air to the DPF. The pressure is measured using a HONEYWELL ABPDANN010BG2A3 pressure sensor (0-10 Bar range), which is connected to an Arduino Uno for data acquisition. The Arduino records pressure readings at 500ms intervals to ensure accurate monitoring. The pressure is gradually increased to a specified value before being released to invert the dome units. The dynamic behavior of the DPF is captured in detail using a Photron Fastcam Mini UX100 high-speed camera, as illustrated in Movie 1. Tip displacement over time is then analyzed using the Tracker Video Analysis and Modeling Tool, providing precise measurements of the system's response.

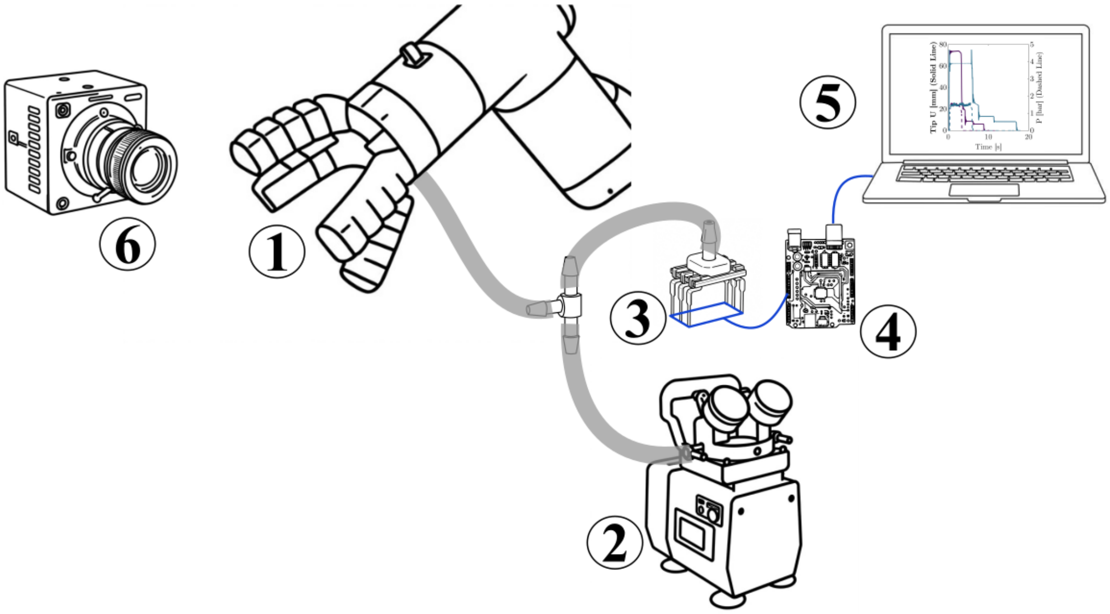

Figure S13: Experimental setup for pressure and displacement measurement. Schematic of the test setup used to record the displacement and pressure over time of the DPF and DFR. (1) DPR or DPF (2) Air Pump. (3) Pressure sensor. (4) Arduino Uno. (5) Laptop. (6) High Photron Speed camera.

A.7.2 Inverse problem experimental validation

Five geometries are tested by activating all bistable unit cells and measuring the final tip position. The final position of the finger is measured by using image processing. Results can be observed in Figure S14a (i-v) for NinjaFlex 85A and Figure S14b (i-iii) for Cheetah 95A, and the target coordinates, model prediction, and experimental measurements are reported in Table S9.

Target Coordinates	Model	Experiments	Error
[46 , 32]	[46.85 , 31.22]	[42.9 , 37.7]	13%
[25 , 20]	[25.34 , 20.34]	[23.53 , 22.93]	10%
[25 , 40]	[30.10 , 40.70]	[29.4 , 43.5]	6%
[25 , 30]	[28.76 , 29.87]	[23.0 , 34.26]	17%
[35 , 45]	[37.98 , 44.36]	[36.8 , 50.77]	11%

Table S8: Experimental measurements for five different DPFs optimized for a specific target position (NinjaFlex 85A).

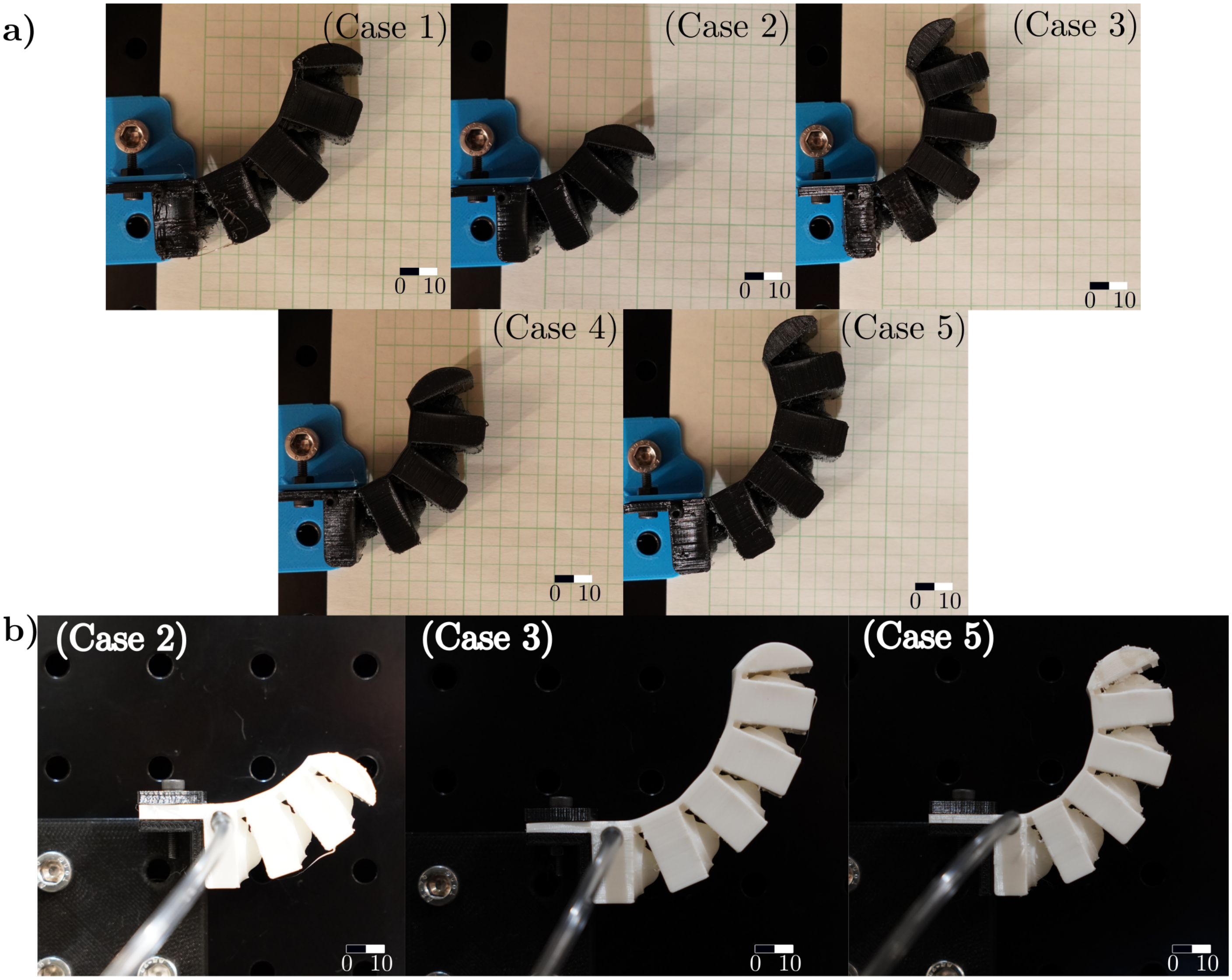

Figure S14: Experimental results for different DPF geometries. a) Printed samples for inverse design target positions (i) - (v) for Ninjaflex 85A b) Printed samples for inverse design target positions (i) - (iii) for Cheetah 95A.

Target Coordinates	Model	Experiments	Error
[46 , 32]	[45.6, 31.8]	[43.31 , 28.38]	6.9%
[25 , 20]	[24.7, 19.5]	[25,17, 16.55]	4.3%
[25 , 40]	[25.15, 39.3]	[23.03, 39.29]	2.5%
[25 , 30]	[25.2, 29.22]	[22.9, 31.6]	1.1%
[35 , 45]	[33.97, 45.3]	[33.35, 44.9]	1.2%

Table S9: Experimental measurements for five different DPFs optimized for a specific target position (Cheetah 95A).

A.7.3 Experimental validation stiffness optimization

The stiffness of each DPF is measured using an Instron universal testing machine and applying a perpendicular force to the tip of the actuator (see Figure S14b). Linear behavior is observed in the neighborhood of each stable state (0, 2, 4, and 5 active units in Figure S14b), with a different stiffness for every state. Finally, the inverse design problem results are tested by comparing the stiffness of two geometries with the same target position but two distinct objective functions (Equation S.30 and Equation 6).

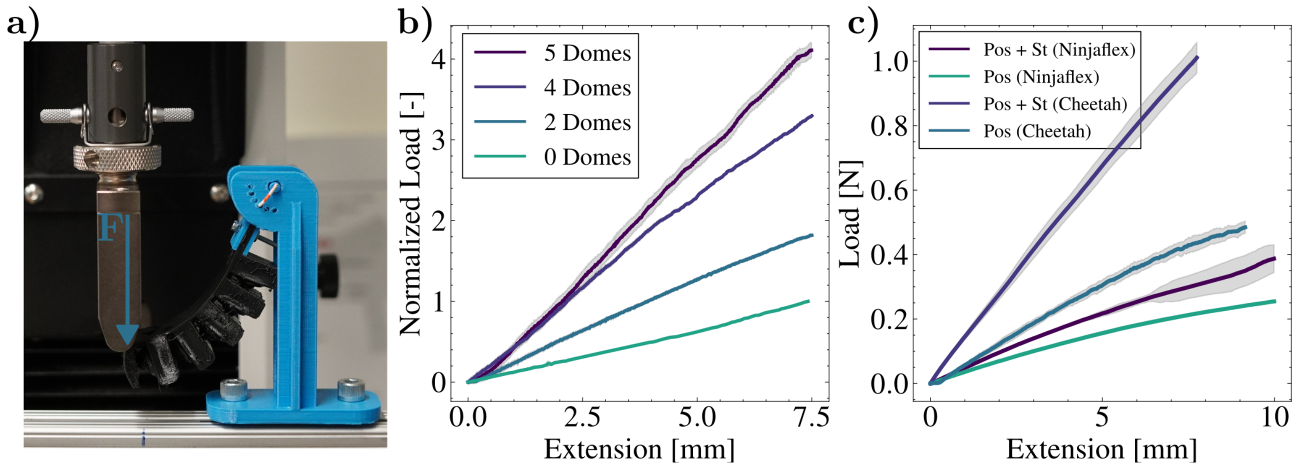

Figure S15: Force vs displacement plot for different DPF. a) Experimental setup. b) Effect of number of active domes on DPF stiffness. c) Comparison between DPF geometries for position inverse design and position+stiffness inverse design for target 5 (Cheetah 95A and NinjaFlex 85A).

A.7.4 Durability Test

The robustness of the DPF design is tested by performing 100 cycles over three weeks to show the geometry's repeatability and effect. Results show that after three weeks and 100 cycles per week, the tip position stays within 5% of the target tip displacement.

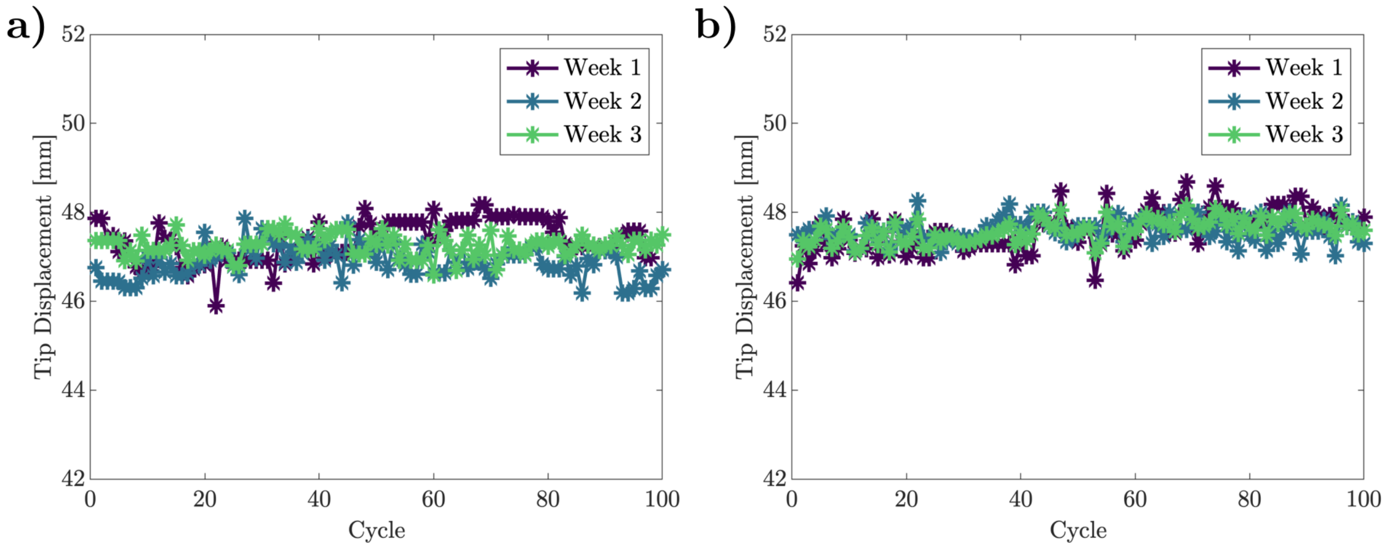

Figure S16: Durability test and time decay response for a) NinjaTek NinjaFlex b) NinjaTek Cheetah (95A).

Notice that the characteristics of multistability reduce the potential effects of material variability by making geometry (i.e., morphology) the dominant driver of our soft robot's response.

A.7.5 Carrying Capacity

Payload capacity and object grasping tests were conducted to evaluate the performance of our DPR, demonstrate its grasping capabilities, and determine the maximum carrying weight of the system. The designed global states enable adjustments in output force and position based on the number of inverted domes (see Movie 5). This adaptability allows the DPR to grasp objects of various sizes and topologies by removing the actuation pressure. Figure S17 illustrates the range of objects successfully grasped by the DPR using only the energy stored in the structure due to dome inversion. As depicted, diverse object types are effectively grasped, highlighting the system's versatility. Additionally, to characterize the payload

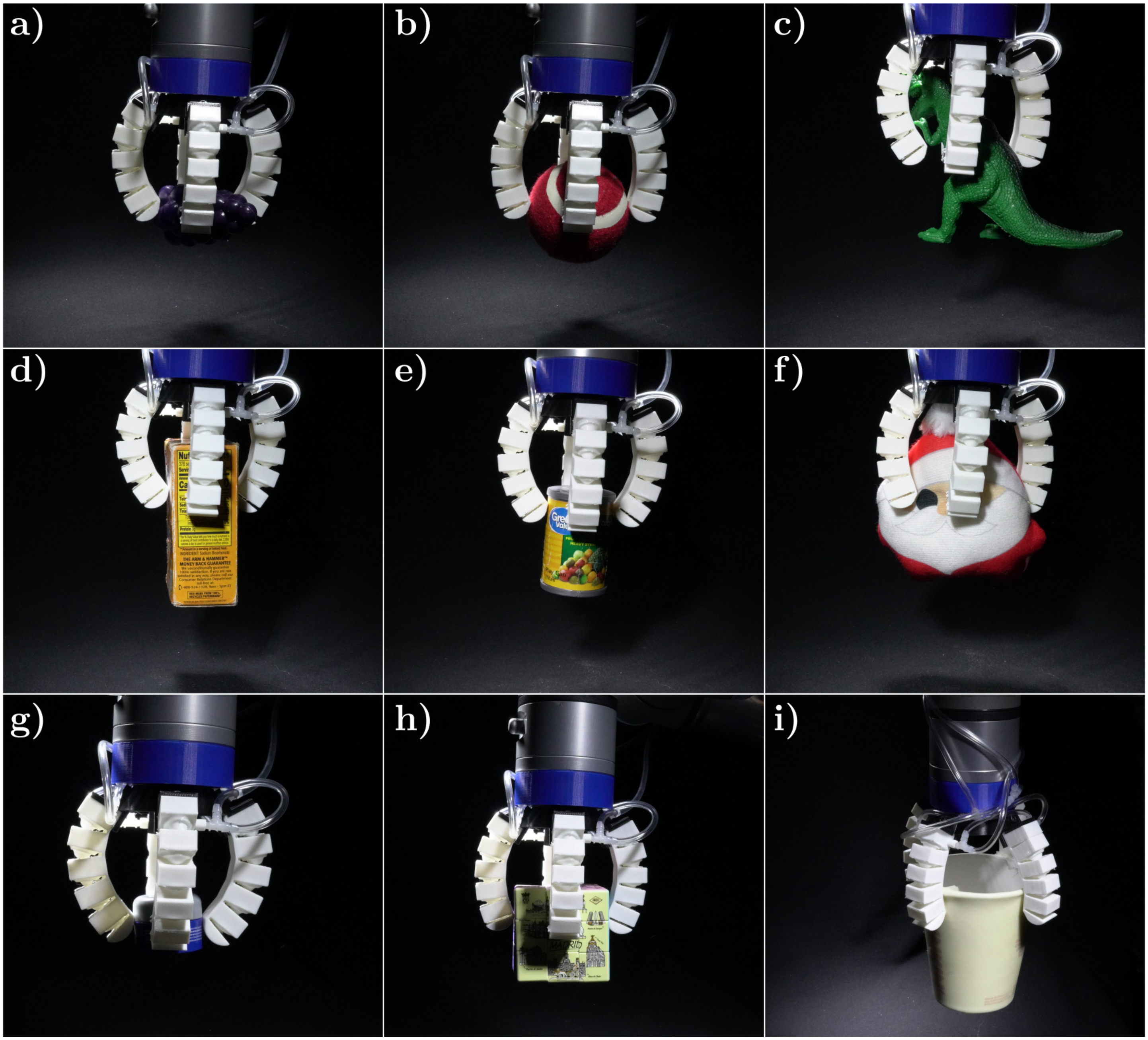

Figure S17: DPF grasping capabilities are shown with six different objects. a) Blackberry Replica, b) Tennis Ball, c) Toy Dinosaur, d) Baking Soda pack, e) Mini Fruit Can. f) Plush Toy g) Cylindrical Cream. h) Rubik's Cube. i) Coffee Cup.

capacity, weights were incrementally added to the object shown in Figure S17 (i) until the structure could no longer grasp it. A maximum load of 738 g was recorded, resulting in a maximum load-to-weight ratio of 10.25 (See Movie 5).

A.7.6 Scalability

As the mechanical response of the DPF is derived from the dome geometry and its stability, the DPF and robot can be scaled to increase to large dimensions just by considering the energy stored during the dome inversion. Following the analysis by Seffen and Vidoli [58], we can derive different additional relations for scaling, given the energy required for inverting a single dome. This energy is proportional to $\frac{Et^3}{1-\nu^2}\left(\frac{H}{R}\right)^2$ where E, ν, t, and R are the elastic modulus, Poisson's ratio, thickness, height, and radius of the domes respectively. We can utilize the dome shallowness $\frac{H}{R}$ and the curvature-to-thickness ratio $\frac{t}{R}$ to either scale up or down the DPF geometry. It should be mentioned that our dome unit bistable behavior can be

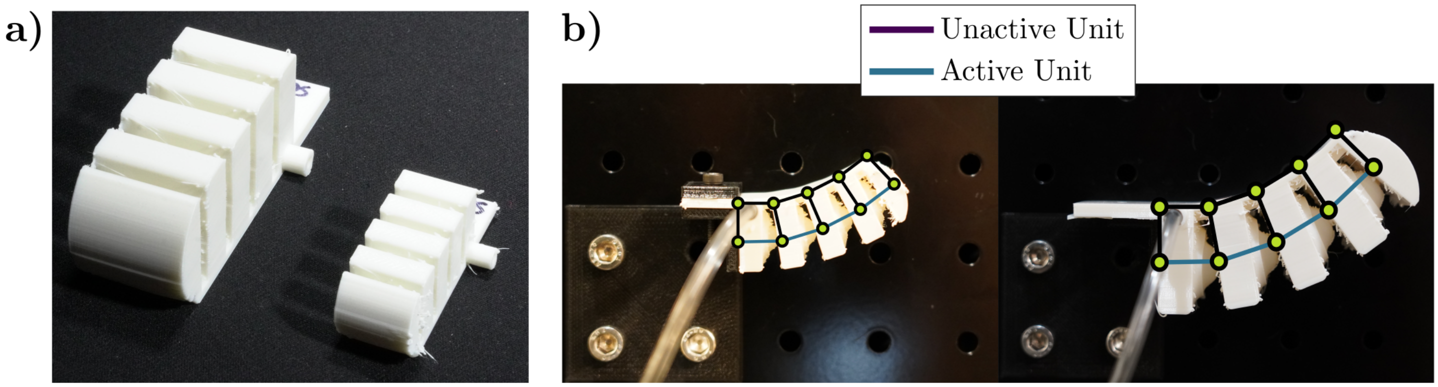

Figure S18: DPF scalability test. a) Two different DPF specimens scale using adimensional relationships. b) Stable states with all dome units active and a comparison with our lattice model.

geometrically scaled; however, for metastable units, it is necessary to consider the effect of the viscoelastic response on a larger system as this response is a combination of the geometry and material response. To test this, we 3D printed two different geometries with the same energetics but a 2x difference.

A.8 Dynamic response for Pick and Place Application

For our pick and place, we create a pressure profile that accurately matches the experimental measurements for our DPF (see Figure S19a). At the same time, the optimization algorithm is utilized to determine the dome heights of the last two units and the remaining geometric parameters (t).

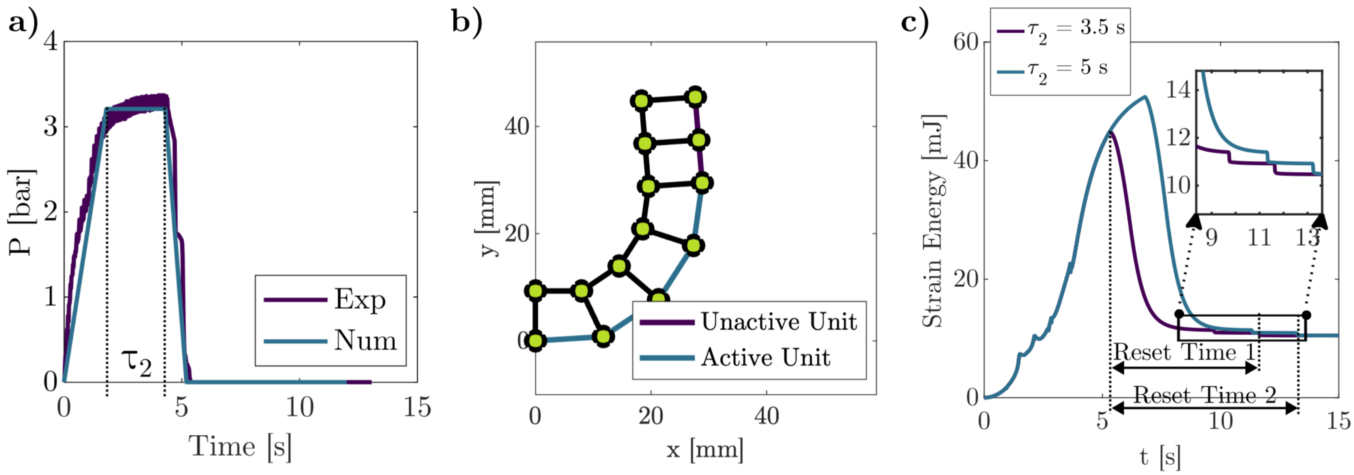

Figure S19: Pick and place task planning set points and model inputs. a) Pressure profile for task planning. b) Setpoint (stable state) for the first active three bistable units. c) Dynamic response for the bistable + metastable actuator.

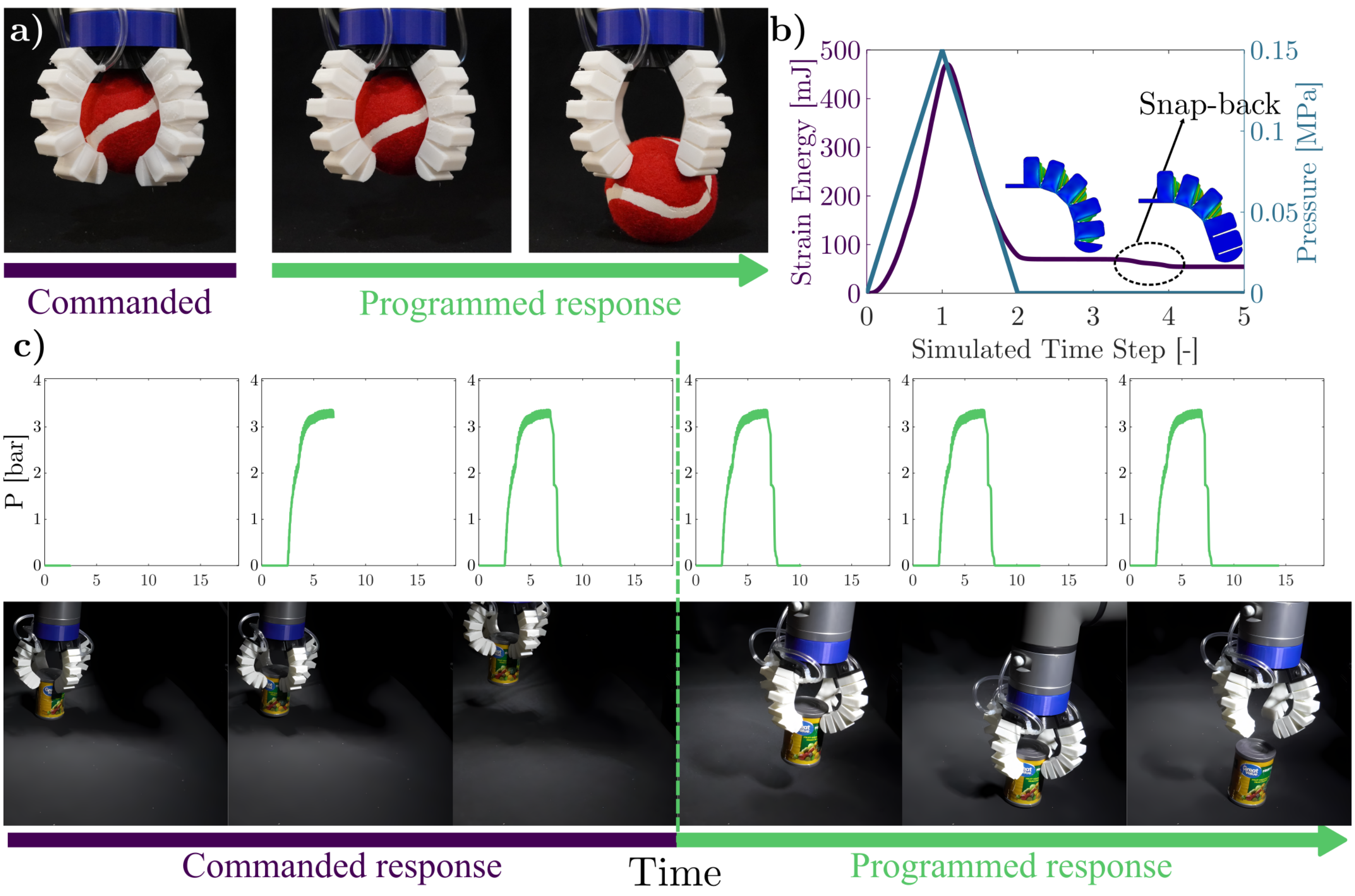

Figure S20: Programmed Pick and Place application for the Dome Phalanx Gripper a) Close up of commanded response when picking a tennis ball. b) Bistable + Metastable finger behavior: Strain energy and pressure vs. time response. c) Snapshots of embodied pick-and place tasks: A single pressure input is given for the robot to pick the object (Commanded response) and then release it depending on the viscoelastic material response (Programmed response)(see Movie 7).

Table S10: Pick and Place DPF final geometry

H1	4.0
H2	4.5
H3	5.0
H4	5.0
H5	3.0
H6	3.0
Unit S	1.0
Unit L	6.0
t	0.83
t_{lim}	1.0
UC	15.00

Given this, a DPG architecture is built that utilizes the dynamic programmed response (see Figure S20a) to perform a simple pick-and-place application after the pressure is released. This behavior is achieved as the metastable domes reset due to visco-elastic effects, showing a snap-back phenomenon on a specific time scale (see Figure S20). It should be mentioned that the metastability can be utilized in either stable state, meaning this embodied task can be performed for different gripper apertures (see Movie 6).

A.8.1 Embodied Classification Task

We leverage the multistability of our DPF to develop a multistable gripper (DPG) capable of achieving multiple aperture configurations. This enables the robot to approximate and adapt to various object sizes. Representative object sizes, corresponding target positions, and optimization results are presented in Table S11, while a visual comparison with the 3D-printed prototypes is shown in Figure S21b. Due to the gripper's symmetry, the aperture is modeled considering only two fingers. By targeting a specific set of aperture values, we encode object size information directly into the gripper's geometry and design. This allows for discrimination between objects with dimensions near the predefined targets. To decode this morphological information, we integrate a contact sensor into one of the finger units (Figure S21c), which detects when the gripper deviates from a stable state. We use a commercial RP-C10-ST pressure sensor (0.2–2 kg), although other sensors capable of detecting perturbations—such as curvature or strain sensors—can also be employed. The sensor threshold is calibrated by observing the sensor reading when the dome behind it is inverted, guaranteeing that all the stable configurations would not be detected as a contact. By utilizing the feedback from the contact sensor and the robot's morphology, we can create a classification loop (see Movie 7) that can classify between different object sizes and weights.

Table S11: Object size and target coordinate for multistable DPR

Object size [mm]	Model
35	35.02
45	45.07
60	60.023
70	73.15

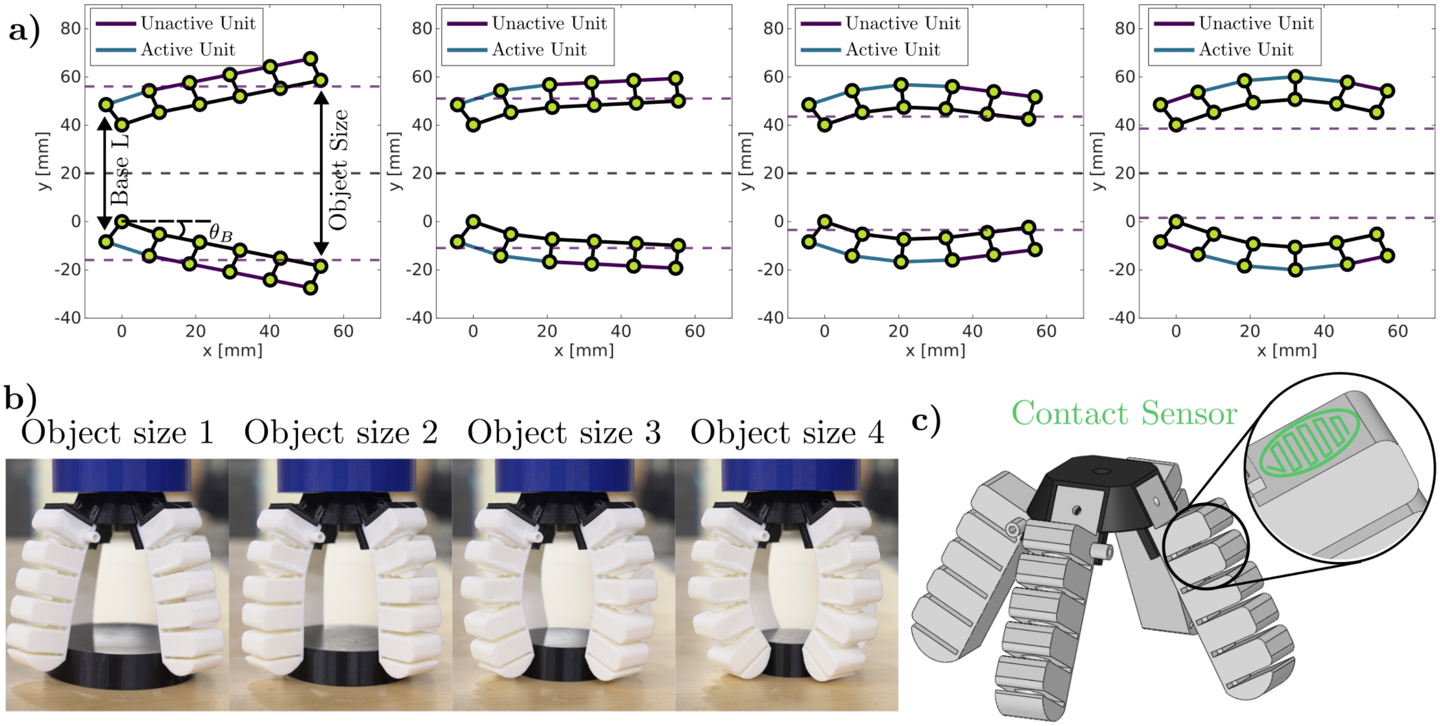

Figure S21: Dome Phalanx Gripper lattice design. a) Lattice model results for the four optimized stable states. b) 3D printed DPG on its four different programmed stable states. Black disks represent the designed object size. c) Contact sensor position placement to detect perturbations from the pre-programmed stable state.

Results for the optimization algorithm for different object sizes can be observed in Table S12.

Table S12: Object classification optimization results

H1	3.81
H2	4.15
H3	4.52
H4	4.82
H5	2.5
Unit S	1.82
Unit L	8.57
t	0.83
t_{lim}	1.05
UC	15.00
Base L	20.0
θ_B	-27.06°

A.9 Dome Phalanx Walker

The dome phalanx walker (Figure S22a) is designed by combining six legs, each composed of different arrangements of dome units (see Figure S22a). The front and back legs are angled at 30°, inspired by leg arrangements observed in insects [59], to enhance both stability and locomotion speed. Each leg has two functional zones (Purple and green zone in Figure S22c), each composed of a combination of different dome unit geometries which can exhibit monostable, metastable, and bistable behaviors. Each leg exhibits two stable states (Figure S22b), enabling the robot to support greater loads while remaining compatible with monolithic 3D printing in its initial flat configuration. To study the leg's motion and design the resetting times of the metastable units, we employ our lattice model to generate a discrete representation of the leg (see Figure S22d). Our central modeling assumption is that units within different planes and zones do not interact, allowing for independent design of each zone while maintaining the model's simplicity. Using this approach, we determine both the metastable units' resetting times and each dome unit's design parameters (Figure S22c) to program the timing of each locomotion phase. Additionally, the model allows us to track the tip displacement in the y-z plane over time (Figure S22e), revealing how metastability amplifies leg movement through the time delays introduced by the viscoelastic response.

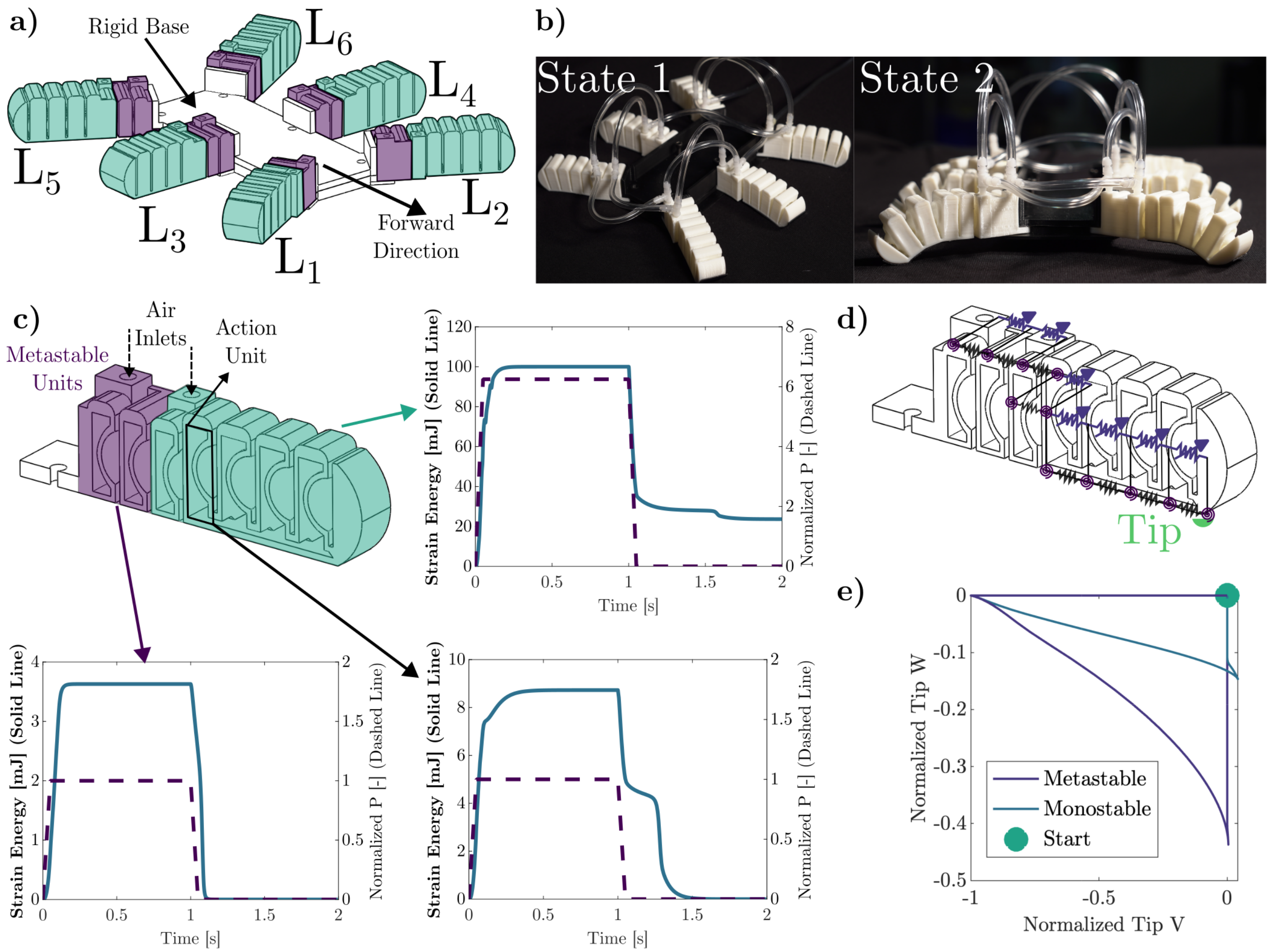

Figure S22: Dome Phalanx Walker design with lattice model. a) DPW architecture. b) Flat state of the walker (State 1) and arc-like state (State 2) by activating the bistable units. c) The DPW leg and its dynamic behavior are divided by zones and units. The metastable action unit exhibits a higher resetting time than the metastable units in the purple region.

The design unit (see Figure S22c) is tuned individually for each leg, enabling versatility and programmability in the walker's behavior. We employ this unit to achieve directional walking by controlling only the actuation pressure (see Figure S23a and b). This is accomplished by introducing a design unit with metastable behavior in the front legs, characterized by a longer resetting time and a higher inversion pressure than the metastable unit used in zone 1 (purple zone).This configuration ensures that the front-leg units do not prematurely revert to their initial state, as actuation pressure is applied before their resetting time elapses. This creates a sustained asymmetry in leg behavior, driving directional movement until the pressure is released or increased on the opposite phase (see Figure S23). It is important to note that the dome geometry directly affects the resetting time—longer resetting times are associated with higher inversion pressures. Accordingly, we designed the front-leg units for a longer resetting time, resulting in higher inversion pressure.

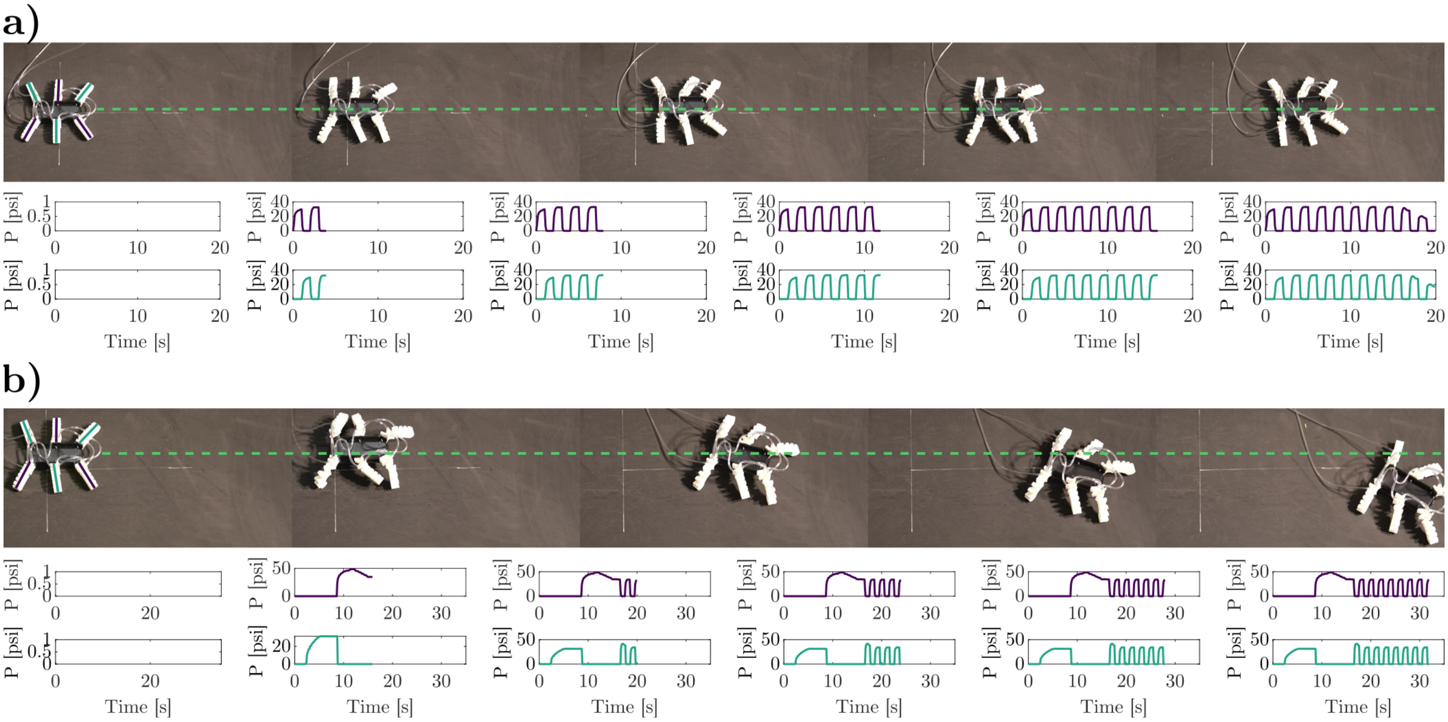

Figure S23: Dome Phalanx Walker programmed response and pressure measurements a) Snapshots of a straight walking direction given a uniform alternating pressure phase. b) Snapshots of the turning maneuver result from an asymmetry created by the pressure increase on the first phase cycle.
